# PbSe-Based Colloidal Core/Shell Heterostructures for Optoelectronic Applications

**DOI:** 10.3390/ma7117243

**Published:** 2014-10-30

**Authors:** Gary Zaiats, Diana Yanover, Roman Vaxenburg, Jenya Tilchin, Aldona Sashchiuk, Efrat Lifshitz

**Affiliations:** Schulich Faculty of Chemistry, Russell Berrie Nanotechnology Institute and Solid State Institute, Technion-Israel Institute of Technology, Haifa 32000, Israel; E-Mails: gzaiats@gmail.com (G.Z.); diana.yanover@gmail.com (D.Y.); romanv@tx.technion.ac.il (R.V.); jtilchin@gmail.com (J.T.); chaldona@tx.technion.ac.il (A.S.)

**Keywords:** PbSe, PbSe/PbS, PbSe/CdSe, colloidal quantum dots, core/shell, alloyed quantum dots, band-gap tunability, surface oxidation

## Abstract

Lead-based (IV–VI) colloidal quantum dots (QDs) are of widespread scientific and technological interest owing to their size-tunable band-gap energy in the near-infrared optical region. This article reviews the synthesis of PbSe-based heterostructures and their structural and optical investigations at various temperatures. The review focuses on the structures consisting of a PbSe core coated with a PbSe*_x_*S_1–*x*_ (0 ≤ *x* ≤ 1) or CdSe shell. The former-type shells were epitaxially grown on the PbSe core, while the latter-type shells were synthesized using partial cation-exchange. The influence of the QD composition and the ambient conditions, *i.e.*, exposure to oxygen, on the QD optical properties, such as radiative lifetime, Stokes shift, and other temperature-dependent characteristics, was investigated. The study revealed unique properties of core/shell heterostructures of various compositions, which offer the opportunity of fine-tuning the QD electronic structure by changing their architecture. A theoretical model of the QD electronic band structure was developed and correlated with the results of the optical studies. The review also outlines the challenges related to potential applications of colloidal PbSe-based heterostructures.

## 1. Introduction

For over a decade, lead chalcogenide (PbTe, PbSe, PbS) colloidal quantum dots (QDs) have been the focus of widespread scientific and technological interest [1,2,3,4]. They are characterized by a rock-salt crystal structure (space group Fm_3m), direct band gaps at four equivalent L-points in the first Brillouin zone, small electron and hole effective masses [5], large dielectric constants [6], and a large effective exciton Bohr radius [7]. Nano-sized IV–VI QDs exhibit size-tunable direct band-gap energy in the range of 0.3–1.7 eV, characterized by a broad-band absorption profile in the near-infrared (NIR)—visible wavelength range [8,9,10]. Moreover, these QDs can be processed by simple low-cost solution-based techniques, readily applicable to fabrication of large-scale devices. The above-mentioned properties of IV–VI QDs make them appropriate for being used in NIR gain devices [9,10], photovoltaic cells (PVCs) [11,12,13,14,15,16,17], Q-switches [18,19], thermoelectric systems [20,21,22], and as biological markers [23,24,25].

The synthesis procedures developed in the past decade made it possible to obtain lead chalcogenide nanostructures with controlled size and morphology [26,27,28,29,30,31,32,33,34,35,36,37,38,39], which laid the grounds for studying their fundamental physical properties, such as the type of conductivity [4], the density of states [40], electron–hole exchange interactions [41], radiative lifetimes [42], Auger relaxation processes, and multiple exciton generation [43,44,45,46,47,48,49,50,51,52,53]. While important results were obtained in the works cited above, the studies devoted to the chemical stability of IV–VI QDs under ambient conditions are still quite scarce [54,55]. At the same time, chemical stability may affect most of the above-mentioned principal QD properties; for example, the photoluminescence (PL) intensity of PbSe and PbS QDs was shown to decrease on their exposure to air [56,57]. In view of the fact that integration of colloidal QDs in functional devices may require fabrication steps under ambient conditions, the issue of the effect of QD chemical stability on their physico-chemical properties should be essential for the currently emerging QD-based technologies. 

Various approaches aimed at reducing the oxidation effect, including controlled passivation of the surface by organic or inorganic ligands, have been proposed [58,59,60]. Several research groups investigated the influence of the cation/anion ratio on the QD surface properties and showed that the excess of Pb^2+^ or Cd^2+^ on the exterior surfaces of IV–VI QDs or II–VI QDs, respectively, may increase the number of bound surfactant molecules, thus improving the surface passivation [61,62,63,64,65]. Other approaches include exchange of the surface ligands controlling the QD properties [65,66]. For example, recent studies reported the immediate influence of oxygen exposure on the luminescence intensity of oleic acid-capped PbSe QDs [55,56,57] and suppression of the oxidation process by exchanging the surfactants for alkylselenide molecules [60]. In other studies, post-synthetic stabilization of the QD surface was achieved by exchanging the organic surfactants for inorganic shells [58] or halide anions [13]. Recently, Bae* et al.* [59] have reported stabilization of colloidal PbSe and PbS QDs as a result of their reaction with molecular chlorine. 

Alternatively, efficient passivation can be achieved by epitaxial growth of another semiconductor layer/s on IV–VI core QDs, leading to the formation of core/shell heterostructures, in which the shell exterior surface remains covered by organic molecules. Another mechanism of the formation of such heterogeneous nanocrystals employs cation exchange, which was demonstrated to occur in various materials such as CdS-PbS [67] and Hg*_x_*Cd_1–*x*_Te [68]. Recently, the formation of PbS/CdS [69,70] and PbSe/CdSe [28,64,71] core/shell QDs by the cation exchange method was also achieved, which marked a progress in solving the problems of luminescence instability and oxidation in these materials. The PbSe/CdSe heterostructures can be further covered with an epitaxial shell of ZnSe(S), which is relatively chemically inert and has high potential barrier [71]. Air-stable QDs were also obtained by encapsulation in metal-oxide compounds (e.g., ZnO) [72].

**Figure 1:**
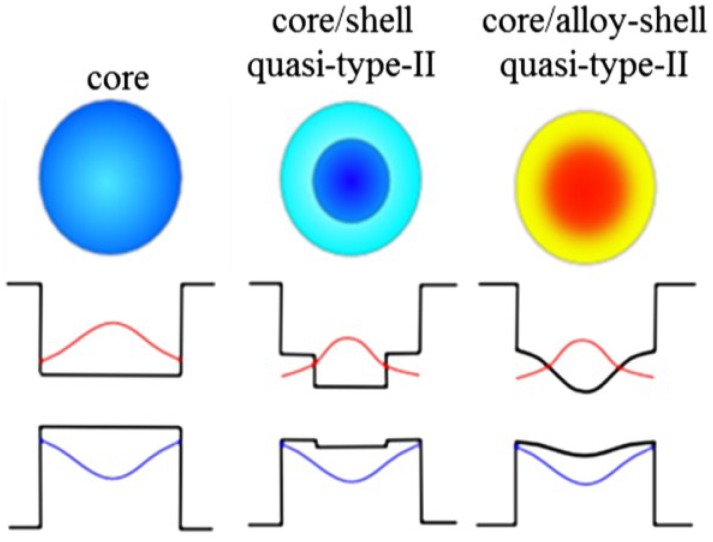
Different-type heterostructures (top row) and possible electron and hole wave-function distributions (bottom row).

As discussed-below, the shell growth not only provides the QDs with chemical and photochemical stability, but may also result in totally new physical properties of the overall heterostructure. Depending on the core-to-shell band-edge offset, different types of heterostructures will be generated. For example, the discussed-above heterostructures have the quasi-type-II alignment, which permits delocalization of one charge carrier over the entire core/shell structure, the other one being confined either in the core or in the shell. In PbSe/PbSe*_x_*S_1–*x*_ (0 ≤ *x* ≤ 1) QDs, the electron is confined in the core, while the hole is delocalized over the entire core/shell heterostructure. This is true for both core/shell and core/alloyed-shell (0 < *x* < 1) QDs, the only difference being the smooth core-shell boundary potential in the latter case [9,73,74]. In contrast to this, in PbSe/CdSe QDs the hole is confined in the core, while the electron is delocalized over the entire core/shell heterostructure. Schematic representation of different heterostructures, along with their anticipated band-edge alignment and the carrier distribution curves, is shown in Figure 1.

The quasi-type-II heterostructure induces partial charge separation, thereby influencing the strength of direct Coulomb and the exchange interactions [75,76]. This, in turn, leads to changes of the radiative lifetime of a single exciton [77] and multiple excitons [78,79,80,81,82], and to their bright-to-dark energy splitting [75,83,84,85].

In recent years, the enhancement of chemical stability as well as tuning of physical properties of colloidal II–VI QDs [86,87,88] and a few types of colloidal IV–VI QDs [31,89] has been successfully achieved in ternary QDs. It should be noted, that our work [9,90] shows the advantage of core/shell and alloyed core/shell heterostructures over core structures. For example, the alloying of PbSe/PbSe*_x_*S_1–*x*_ results in smoothing the core-to-shell boundary potential (see Figure 1) and in considerably enhanced chemical and photochemical stability [9,73,77]. An epitaxially grown PbS or PbSe*_x_*S_1–*x*_ shell has an extremely small crystallographic mismatch (≤3%) with the PbSe core, thereby reducing the core/shell interface defects [9,90]. Recently developed alloyed QD heterostructures, such as Cd*_x_*Zn_1–*x*_Se/ZnSe [91], CuInS_2_/ZnS [92], and CdTe/CdTe*_x_*Se_1–*x*_ [62,93], show extremely high spectral stability (blinking-free QDs) [62,93] and a relatively long biexciton lifetime (0.5 ns) [94]. We have recently reported a theoretical study of the properties of alloyed PbSe/PbSe*_x_*S_1–*x*_ core/shell colloidal QDs, which shows the variability of the electronic properties in all alloyed heterostructures with elemental composition, modification of the exciton-phonon interaction, direct Coulomb and exchange interactions [74,90,95]. Additional details of this one and the other recent theoretical work are presented below. Since the requirements for the QDs to be used in any optoelectronic device include size uniformity, high-yield chemical synthesis, nearly defect-free structure, efficient absorption, and photochemical stability, the review also describes different methods of synthesis PbSe/PbSe*_x_*S_1–*x*_ (0 ≤ *x* ≤ 1) and PbSe/CdSe heterostructures [73,96], whose electronic structure and optical properties were investigated [71,97,98].

This article is organized in the following way. In Section 2, two approaches for modeling the electronic-band structure of the above-mentioned heterostructures are described. The first approach employs the multiband **k•p** envelope function for the description of PbSe/PbSe*_x_*S_1–*x*_ core/shell QDs. The model shows the existence of the quasi-type-II electronic configuration, which depends on the QD size, internal architecture, and shell composition. The second approach, which employs the finite element analysis (FEA) software (COMSOL), was applied for evaluating the effective mass model of PbSe/CdSe core/shell QDs. These models help to get qualitative understanding of QD-related physical phenomena and possible ways of engineering electro-optical properties of QD heterostructures. Section 3 summarizes the currently applied methods of QD synthesis, which include epitaxial growth and cation-exchange as well as the crystallographic, morphological, and chemical characteristics of PbSe-based core/shell heterostructures. Section 4 describes the steady-state and time-resolved optical properties of the QDs under consideration, measured at various temperatures, with or without exposure to oxygen. In this section, the optical properties of different-type QDs are compared and certain particularities are discussed. Besides, the possibility of tuning the QD band-edge optical properties by changing their composition and architecture is considered. The results demonstrate that the produced PbSe/PbSe*_x_*S_1–*x*_ (0 ≤ *x* ≤ 1) and PbSe/CdSe heterostructures are characterized by relatively narrow emission bands, longer excited-state lifetimes, and higher stability towards oxidation for a restricted period of time. Finally, in Section 5, several applications of the discussed above PbSe-based heterostructures are considered.

## 2. Theoretical Prediction of the Electronic Properties of QD Heterostructures

Several calculation approaches, such as **k•p** [95,99], effective mass [100], tight binding [101], and pseudopotential [83,84] methods, were successfully applied for describing the electronic properties of QDs. Recently, the effective mass method was used in combination with the FEA software (COMSOL) [102], which proved to be a convenient qualitative way of approximating a wide variety of geometrical shapes, which cannot be treated analytically [102].

### 2.1. Electronic Structure of PbSe/PbSe_x_S_1–__x_ (0 ≤ x ≤ 1) QDs

The electronic-band structure of QDs (both alloyed and without alloying) was evaluated using the four-band **k•p** envelope function method [40], which is based on solving the effective Schrödinger equation, ${\hat{H}}_{k\cdot p}\left(-i\nabla \right)F\left(r\right)=EF\left(r\right)$ , where **F**(**r**) are the four-component envelope eigenfunctions. The model considered such specific features as the effective mass discontinuity, the crystal potential, and the dielectric constant of the constituents at the core/shell and at the shell/surrounding interfaces [95], in the cases when the shell has the alloy composition. A PbSe/PbSe*_x_*S_1–*x*_ (0 ≤ *x* ≤ 1) core/alloy-shell QD is schematically represented in 2a, where *R*c and *R*s are the core and shell radius, respectively, and *W* is the shell thickness. Hamiltonian ${\hat{H}}_{k\cdot p}\left(-i\nabla \right)$ was adjusted to account for the effective mass discontinuity at the PbSe/PbS core/shell QD interface by an appropriate choice of the kinetic-energy term, which ensured the probability current conservation and the envelope functions continuity [103].

Furthermore, a few other issues were addressed in this theoretical study, namely, the effective mass anisotropy (which is typical of IV–VI semiconductors), expressed via the remote band contributions to the longitudinal (∥) and transverse (⊥) conduction (−) and valence (+) band effective masses, ${\text{m}}_{||,⊥}^{\pm }$; the dependence of each physical parameter on the radial position (*r*) across the dot (2b), and its smooth variability across the core/shell and the shell/surrounding interfaces. The smooth potential profile reflects the nature of the interface region in alloyed materials with a gradient composition. The overall band offset was chosen to be the same as in the corresponding bulk materials (in bulk PbS, the valence-band maximum lies 0.025 eV above that in bulk PbSe and the conduction-band minimum lies 0.155 eV above that in bulk PbSe). The previously calculated PbS band offset relative to that of PbSe [104] and the reported PbS bulk electron affinity [105] and the composition-dependent bulk PbSe*_x_*S_1–*x*_ band-gap energy [106] were used. The vacuum level was selected as the zero reference energy. The height of the outer barrier (at *r* = *R*_S_) for both electrons and holes was assumed to be equivalent to the corresponding bulk electron affinity. The carriers mass outside the QD was supposed to be equal to the free electron mass. Under the above assumptions, the values of the conduction and the valence band-edge energies of the QDs can be obtained from the following expressions: $V^{-}=-\left(4.6+0.155x\right)eV$ and $V^{+}=-\left(5.01+0.025x\right)eV$ , respectively.

**Figure 2:**
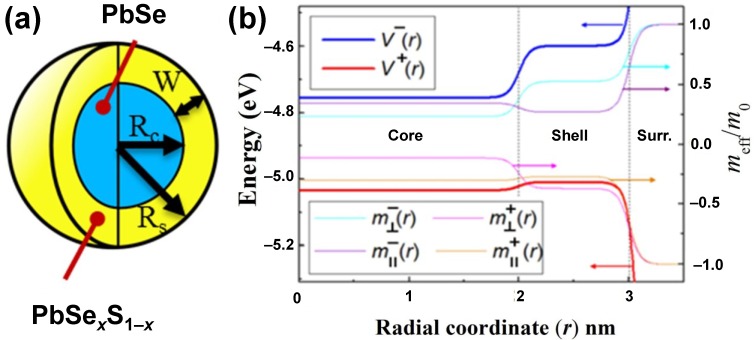
(**a**) Schematic representation of a QD heterostructure with alloyed-shell composition and general chemical formula PbSe/PbSe*_x_*S_1–*x*_, where 0 ≤ *x* ≤ 1. *R*c and *R*s are the core and shell radius, respectively, and *W* is the shell thickness; (**b**) P lot of the conduction and valence band-edge energies, V^−/+^ (left axis), and the remote band contributions to the valence and conduction band effective masses, ${\text{m}}_{||,⊥}^{\pm }$ , in the units of free electron mass, m_0_ (right axis),* vs.* the radial coordinate in the heterostructure shown in (**a**), with *R*c = 2 nm and *Rs* = 3 nm. The zero energy is taken at the vacuum level.

The obtained typical dependences of the effective masses and band-edge energies on the radial position are illustrated in 2b. It should be noted that the influence of the ligands and of the surrounding solvent may lower the barrier height to a certain limited extent, but the general trends shown in Figure 2b remain unchanged. The investigated QDs were the core or core/(alloyed-)shell structures with a general formula of PbSe/PbSe*_x_*S_1–*x*_, where the cases of *x* *=* 1, *x* = 0, and 0 < *x <* 1 correspond to a simple core (PbSe), a core/shell (PbSe/PbS), and a core/alloyed-shell structures, respectively. It follows from the theoretical considerations that effective extraction of both charge carriers, which is required, e.g., in PVC devices, is possible on their penetration into the surrounding medium.

The step-like band alignment of the core/shell alters the confining potential that acts on the charge carriers. The plots presented in Figure 2b show that the bulk band offsets between the core and the shell materials depend on their composition. Moreover, for relatively small QDs, the typical confinement energies are much larger than the band offset energies. Consequently, the lowest electronic levels lie above the band offset and thus are only weakly affected by the core/shell architecture. However, in sufficiently large core/shell QDs, the confined electronic levels are located below the offset energy, which results in the localization of at least one carrier within the core. In contrast to this, the hole is less affected by the small valence-band offset and remains more delocalized as the QD size changes. Since the band offset is a function of composition and the confinement energy is a function of size, the degree of the electron localization in the core (or, equivalently, its location within the system) can be controlled by varying the above two degrees of freedom.

The extent of the electron and hole separation in PbSe/PbSe*_x_*S_1−*x*_ heterostructures can be further analyzed by evaluating the probability of finding a carrier within the core, *P*_core_, or within the shell, *P*_shell_:

(1) $$P_{core}^{e(h)}=\int_{core}\sum_{j=1}^{4}{\left|F_{j}^{e(h)}\left(r\right)\right|}^{2}d^{3}r$$

(2) $$P_{shell}\approx 1-P_{core}$$

**Figure 3:**
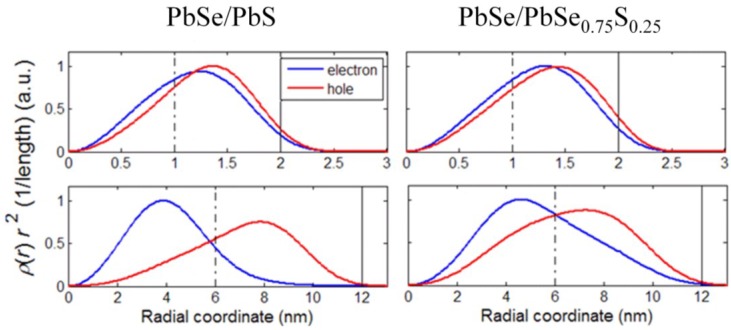
Radial probability density of the ground-state electron and hole in the indicated QD heterostructures. Top row: *R*c = 1 nm, *R*s = 2 nm; bottom row: *R*c = 6 nm, *R*s = 12 nm. Solid and dashed vertical lines designate the external QD boundaries and the core/shell interface positions, respectively.

Figure 3 shows the radial probability density of the ground-state electron and hole in the PbSe/PbS, PbSe/PbSe_0.75_S_0.25_ heterostructures with *R*_C_ = 1 nm, *R*_S_ = 2 nm (top row) and *R*_C_ = 6 nm, *R*_S_ = 12 nm (bottom row). It can be seen that the electron-hole charge separation becomes more pronounced with the growth of the overall QD size at a constant *R*_S_*/R*_C_ ratio. In addition, charge separation appears to be slightly more efficient in PbSe/PbS as compared to alloyed PbSe/PbSe_0.75_S_0.25_ QDs because of larger band offsets in the former structure (see Figure 3, bottom row). 

**Figure 4:**
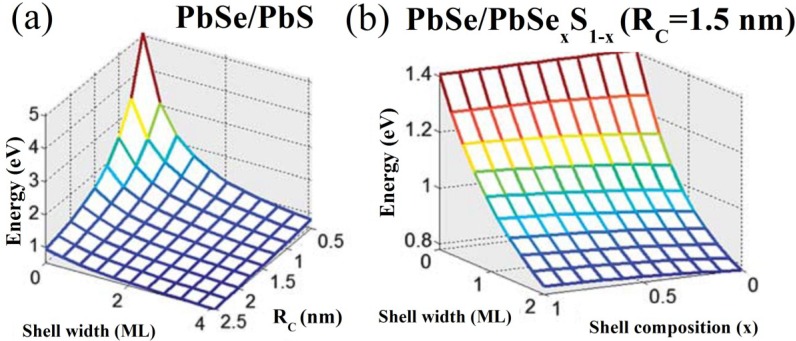
Variation of the ground-state exciton energy, *Eg*, with (**a**) core radius, *R*c, and shell thickness, *W*, in PbSe/PbS QDs, and (**b**) shell thickness, *W*, and composition, *x*, in PbSe/PbSe*_x_*S_1–*x*_ core/alloyed-shell QDs with *R*c = 1.5 nm. ML stands for “monolayer”.

The value of the ground-state exciton energy (*E*_g_) is of major practical and experimental importance. The dependence of *E*_g_ on *R*_C_ and *W* in PbSe/PbS core/shell QDs (see Figure 4a) shows that the *E*_g_ values in core/shell QDs are smaller than those in pure core QDs of the same overall size, *R*_S_, which is in agreement with previous experimental observations [90]. The dependence of *E*_g_ on *W* and *x* in PbSe/PbSe*_x_*S_1–*x*_ core/alloyed-shell QDs with *R*_C_ = 3 nm, presented in Figure 4b demonstrates the possibility of controlling the tunability of *E*_g_ not only by varying the core/shell size, but also by changing the QD-shell composition. Thus, the described above diagrams can be used as a practical tool of predicting the core/shell design for a required ground-state exciton energy.

### 2.2. Electronic Structure of PbSe/CdSe QDs

**Figure 5:**
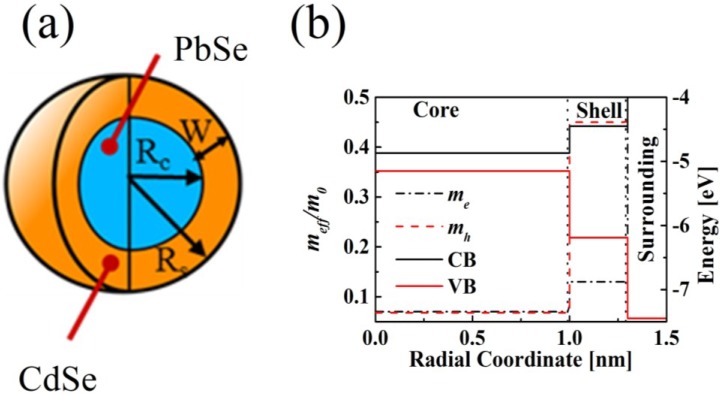
(**b**) Dependencies of the energy bands (VB, CB) and electron and hole effective mass (*m*_e_ and *m*_h_, respectively) values versus radial coordinate in the PbSe/CdSe QD schematically represented in (**a**).

The effective mass approach [102] was implemented using the FEA software (COMSOL) to calculate the wavefunction distribution of charge carriers and the relevant eigenvalues. There is some disagreement in the literature regarding bulk band alignment and effective masses of PbSe and CdSe [100,102,107]. The band alignment and the effective mass values that were used in this work are shown in Figure 5b and correspond to the PbSe/CdSe core/shell heterostructure illustrated schematically in Figure 5a with the same designations as in Figure 2a. Pronounced discontinuity in the hole characteristic properties can be observed at the point of transition from the PbSe core to the CdSe shell, while, in the case of electron, these changes are much smaller. 

**Figure 6:**
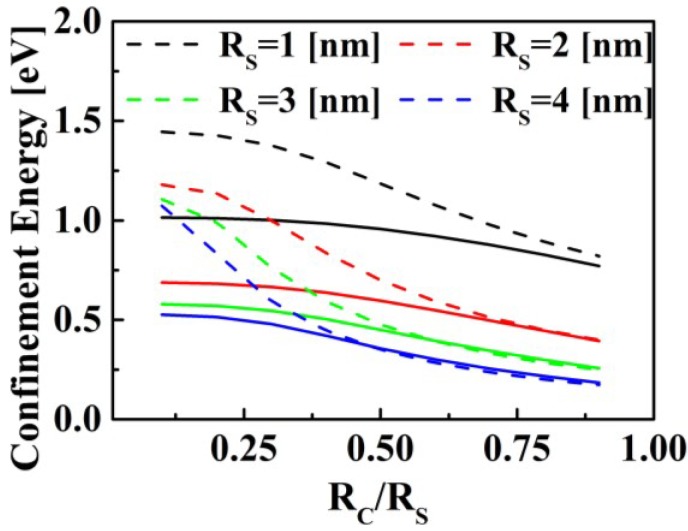
Effect of total QD radius, *R*_S_, and core-to-shell radius ratio, *R*c*/R*s, on the confinement energy of electron (solid lines) and hole (dashed lines).

The curves in Figure 6 show the effect of the total QD radius, *R*_S_, and the core-to-shell radius ratio, *R*_S_*/R*_C_, on the confinement energy of a hole and an electron. As expected, for both types of charge carriers, the *R*_S_ reduction results in the modification the confinement energy and, consequently, of the ground-state exciton energy, *E*_g_, in the QDs. Since the effective masses of an electron and a hole (*m*_e_ and *m*_h_, respectively) in PbSe core have similar values, their characteristic confinement energies become very close as the *R*_C_ value approaches that of *R_S_*. The effective mass of the hole in CdSe shell is a magnitude of order larger than that in PbSe. Therefore, in PbSe/CdSe QDs, the confinement of the hole is larger in the core. Thus, the hole is expected to be localized in the core, while the electron should be delocalized over the entire QD volume. However, in thin shell PbSe/CdSe QDs the penetration of both charge carriers close to the QD exterior surfaces would enable their effective extraction in the PVC configuration.

**Figure 7:**
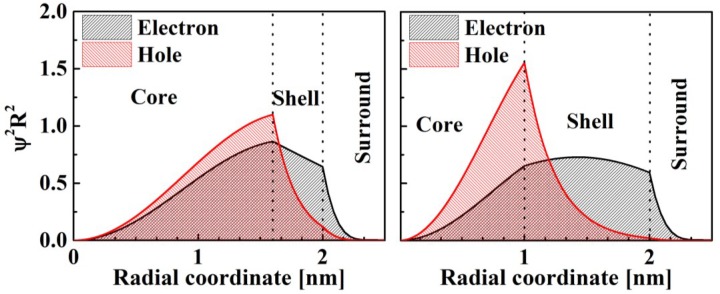
Probability distribution of charges in QD heterostructures with external radius *R_S_* = 2 nm and different core radii: (**a**) *R*_C_ = 1.6 nm; (**b**) *R*_C_ = 1.0 nm.

Figure 7 shows the normalized ( $\int_{QD}{\text{ψ}}^{2}R^{2}=1$ ) probability distribution of the charges in heterogeneous PbSe/CdSe core/shell QDs and in the surrounding medium. Since the confinement energy of the hole is higher than that of the electron (Figure 6). Therefore, the electron is delocalized from the core towards the shell, while the probability of finding the hole in the shell decays exponentially as the shell thickness increases. As a result, the hole is localized inside the core (Figure 7).

The shell thickness is an essential parameter for controlling the electro-optical properties of quantum dots. However, if the shell thickness is chosen so as to provide optimal charge separation, the probability of finding the hole at the QD edge approaches zero (Figure 7b). Thus, employing the core-shell approach for engineering the electro-optical properties of QDs requires careful examination of the wave-function distribution in the particular heterogeneous structure.

## 3. Synthesis, Structural, and Compositional Characterization of PbSe/CdSe and PbSe/PbSe*_x_*S_1–*x*_ (0 ≤ *x* ≤ 1) Colloidal Core/Shell QD Heterostructures

PbSe/PbSe*_x_*S_1–*x*_ (0 ≤ *x* ≤ 1) and PbSe/CdSe core/shell QD heterostructures were formed by one- or two-step procedures (epitaxial growth or cation-exchange synthesis), carried out at various temperatures. The colloidal QD morphology, crystalline structure and size were examined by the methods of high-resolution transmission electron microscopy (HRTEM), scanning tunneling microscopy (STM), and powder X-ray diffraction (XRD). Elemental analysis was performed by means of X-ray photoelectron spectroscopy (XPS). The QD growth and shell coating was monitored by absorption measurements.

### 3.1. Small-Sized PbSe/PbS Core/Shell Colloidal QDs with Band-Gap Energy in the Range of 1.0–1.4 eV

The synthesis and characterization of small-sized PbSe and PbSe/PbS core/shell QDs were described previously [108]. In brief, the preparation procedure included two steps. First, small-sized PbSe core QDs, 2.0–2.5 nm in diameter, were synthesized. The main reaction mixture was composed of lead oleate (Pb(OA)_2_) and hexadecane (HDC). The selenium precursor solution, containing trioctylphosphine (TOP), diphenylphosphine (DPP), and hexadecane (HDC), was heated under vacuum for one hour. Then, this solution was injected into the main reaction mixture and the QDs produced were separated by centrifuging. At the second step, the shell growth was carried out. Diluted bis(trimethylsilyl) sulfide (TMS_2_S) was added dropwise, at 70 °C, into the reaction mixture containing PbSe QDs, Pb(OA)_2_, and diphenylether (DPE), and the obtained core/shell QDs were separated by centrifuging. 

**Figure 8:**
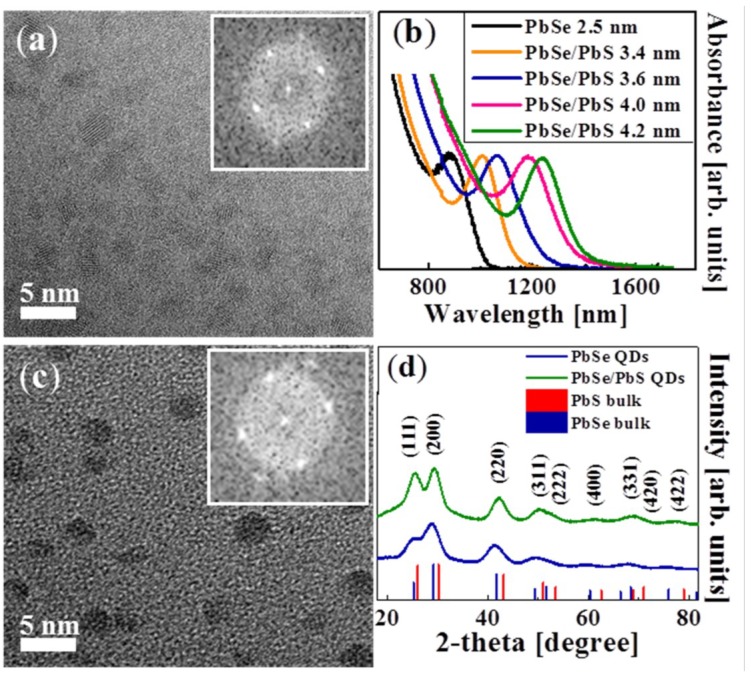
(**a**) HRTEM image of 2.5 ± 0.4 nm PbSe QDs and its FFT pattern (inset); (**b**) absorption spectra of the same PbSe and PbSe/PbS QDs with the same core diameter as in (**a**) and with different shell thickness and the total diameter (see the legend); (**c**) HRTEM image of 3.5 ± 0.5 nm PbSe/PbS QDs and its FFT pattern (inset); (**d**) XRD patterns of PbSe (blue line) and PbSe/PbS (green line) QDs indexed to the bulk rock-salt crystal structures of PbSe (blue vertical lines) and PbS (red vertical lines).

An HRTEM image of PbSe QDs with the average diameter of 2.5 ± 0.4 nm is displayed in Figure 8a. The corresponding fast Fourier transform (FFT) pattern, shown in the inset, confirms full crystallinity of the QDs. Figure 8b demonstrates a set of the absorption spectra of PbSe QDs and of the corresponding PbSe/PbS heterostructures with variable shell thickness (the total QD diameters are indicated in the legend). The growth of a PbS shell leads to a pronounced red shift of the PbSe/PbS QD absorption spectra as compared to those of the PbSe QDs. An HRTEM image of PbSe/PbS QDs with the core diameter of 2.5 ± 0.4 nm and the shell thickness of ~0.5 nm is displayed in Figure 8c, the corresponding FFT pattern being presented in the inset. 

Figure 8d shows the XRD patterns of PbSe and PbSe/PbS QDs. The (111), (200), and (220) diffraction peaks, which appear at 2θ equal to 25.44°, 29.21°, and 41.80°, respectively, confirm the rock-salt structure of both the PbSe and the PbSe/PbS QDs. The diffraction patterns of the corresponding bulk PbSe and PbS are designated by the vertical lines in the figure. Based on the XRD patterns, the average lattice parameters (*a*) in PbSe core and in core/shell PbSe/PbS QDs were calculated to be 6.15 ± 0.04 Å and 6.07 ± 0.03 Å, respectively. The comparison with the bulk values (*a* = 6.12 Å and 5.94 Å for PbSe and PbS, respectively) shows that the core/shell lattice parameter has an intermediate value between the bulk parameters of its constituents.

**Figure 9:**
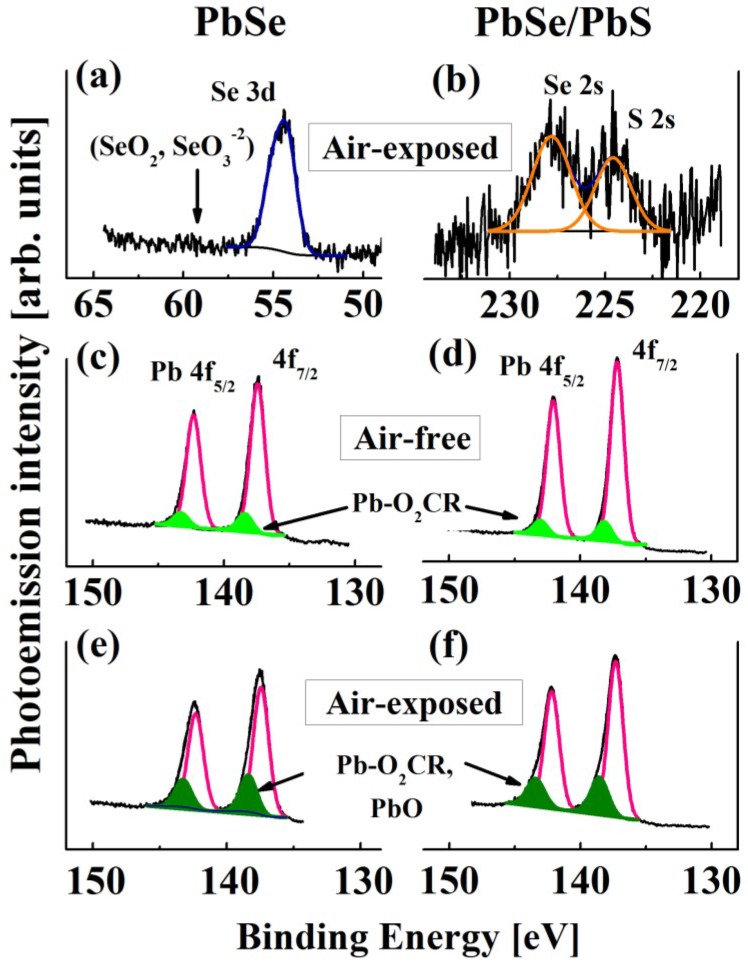
(**a**) XPS spectra of Se 3d peak (dark-blue) for air-exposed PbSe QDs. The location the oxidized-Se peaks (SeO_2_, SeO^3−^) is marked by an arrow; (**b**) XPS spectra of Se 2s and S 2s peaks for air-exposed PbSe/PbS QDs; (**c**,**d**) XPS spectra of Pb 4f peak of air-free PbSe and PbSe/ PbS QDs; (**e**,**f**) XPS spectra of Pb 4f peaks of the same QDs as in panels C and D after 50 min of air exposure. In (**c**–**f**) the curve-fitted individual contribution of Pb−Se/S and of Pb−O bonding is labeled in pink and green, respectively. The diameters of the examined PbSe and PbSe/PbS QDs were 2.5 ± 0.4 nm and 3.5 ± 0.5 nm, respectively.

Representative XPS spectra of air-free/air-exposed PbSe QDs (2.5 ± 0.4 nm in diameter) and of PbSe/PbS QDs (3.5 ± 0.5 nm in diameter) are shown in Figure 9. The 4f_5/2_ and 4f_7/2_ spin-orbit splitting of the Pb 4f level in air-free PbSe and PbSe/PbS QDs are shown in Figure 9c,d, respectively. Both the 4f_5/2_ and 4f_7/2_ peaks were additionally resolved into two doublets each, one doublet corresponding to the lead-chalcogen bond (Pb–Se/S; pink) and the other one corresponding to the lead-oxygen bond (Pb–O; green), which originates from the surface lead ions bound to the oleic acid ligands (–O_2_CR). The binding energies (BEs) corresponding to the XPS peaks are summarized in Table 1. Figure 9e,f shows the XPS spectra of the same QDs as in Figure 9c,d, after 50 min of air exposure. Here, both the 4f_5/2_ and 4f_7/2_ doublets were also resolved into two additional doublets, one of them corresponding to the lead-chalcogen bond (Pb–Se/S; pink) and the other one corresponding to the lead-oxygen bond (Pb–O; dark green), which originates from the surface Pb ions bound to the oleic acid ligands (–O_2_CR) and also from the oxidized surface Pb ions, e.g., from PbO. The comparison of the light-green and the corresponding dark-green areas of the PbSe and PbSe/PbS QD peaks, respectively, shows a ~three-fold increase of the PbO-related signal in the air-exposed QDs as compared to the air-free QDs.

However, in the Se 3d spectrum of the air-exposed PbSe QDs (see Figure 9a) there is no signal (the expected location, ~59 eV, is indicated by an arrow) produced by Se^4+^ (as part of the SeO_2_ or SeO_3_^2−^ species), which could arise from Se oxidation. This fact can be explained by high non-stoichiometry, associated with the Pb-cation-rich exterior surface of the small-sized QDs [108,109,110]. The S 2s and Se 2s peaks of the air-exposed PbSe/PbS QDs are shown in Figure 9b. Since the noise level is high, the signal of oxidized S 2s (~234 eV) cannot be unambiguously identified here, which makes it reasonable to assume that PbSO_3_ species may also exist on the QD surface [55,108].

**Table 1:** Binding energies (BEs) corresponding to the XPS peaks of the PbSe and PbSe/PbS QDs as in Figure 7.

Types	Air-free		Air-exposed				
	Pb 4f_7/2_ (Pb-Se/S) BE	Pb 4f_7/2_ (Pb-O) BE	Pb 4f_7/2_ (Pb-Se/S) BE	Pb 4f_7/2_ (Pb-O) BE	Se 3d BE	Se 2s BE	S 2s BE
**PbSe**	138.42	139.38	138.67	139.61	227.80	–	–
**PbSe/PbS**	137.18	138.16	137.31	138.55	–	227.80	224.58

### 3.2. Large PbSe/PbSe_x_S_1–x_ Core/Shell Colloidal QDs with the Band-Gap Energy in the Range of 0.62–1.0 eV

**Figure 10:**
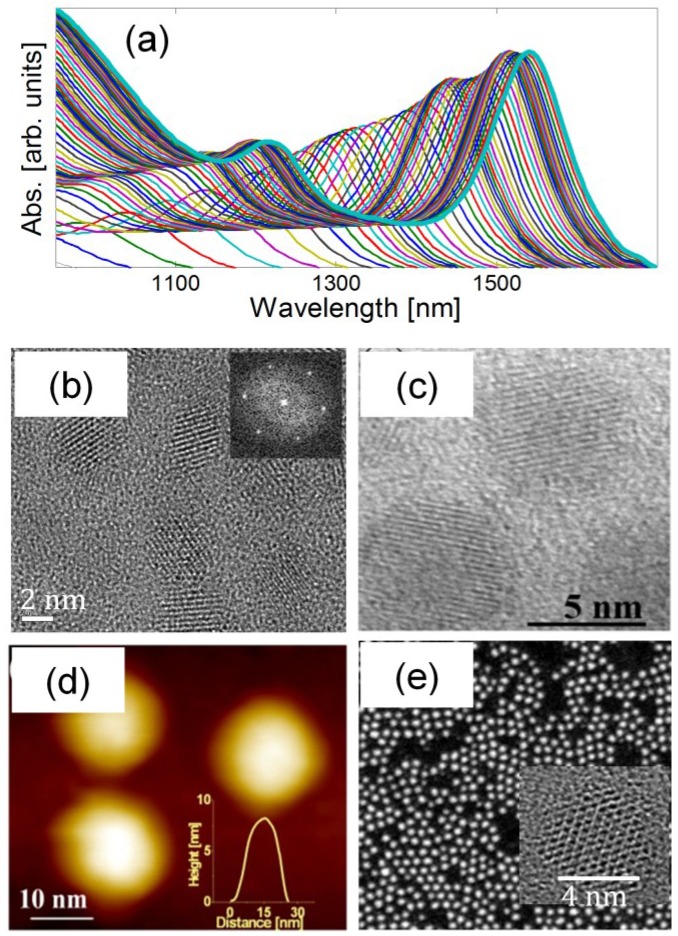
(**a**) Representative absorption spectra of PbSe QDs monitored* in situ* during their synthesis and recorded with a time interval of 10 s; (**b**) HRTEM image of PbSe/PbS QDs; *R*s*/R*c = 2.1/1.5 nm. Inset: FFT pattern of the same QDs; (**c**) HRTEM image of PbSe/PbS QDs; *R*s*/R*c = 3.0/1.5 nm; (**d**) STM image of PbSe/PbS QDs; *R*s*/R*c = 4.0/1.5 nm. Inset: Height profile of a single QD; (**e**) STEM image of PbSe/PbSe*_x_*S_1–*x*_ core/alloyed-shell QDs; *R*s*/R*c = 2.5/1.5 nm. Inset: HRTEM image of a single QD.

The synthesis of large-sized (4.2–10 nm) PbSe/PbSe*_x_*S_1–*x*_ (0 ≤ *x* ≤ 1) core/shell colloidal QDs with the tunable band-gap energy in the range of 0.62–1.0 eV is described in details elsewhere [9], so, in what follows, only a brief description is given. The preparation of core/shell PbSe/PbS included two steps. At the first stage, PbSe cores were produced and purified. The cores were prepared by injecting TOPSe into the main reaction mixture, containing Pb(OA)_2_ and DPE, at 180 °C, while the growth proceeded at 120 °C [3,111,112]. The absorbance curves recorded* in situ* during the QD growth, with time intervals of 10 sec, are shown in Figure 10a. The spectra are characterized by the first 1S_e_–1S_h_ transition (the energy ranging from 1.2 to 0.8 eV), which originates from PbSe QDs with diameters between 1.5 ± 0.2 and 2.6 ± 0.2 nm [110,113]. At the second stage, PbS shells were epitaxially grown on the PbSe cores by the injection of Pb(OA)_2_ and TOPS, at 120 °C, into the reaction solution containing PbSe QDs in DPE. The PbS shell, 1–2 monolayers (MLs) thick, was produced within the first 15–30 min of the reaction.

An HRTEM image of PbSe/PbS core/shell QDs, with *R*_S_*/R*_C_ = 2.1/1.5 nm, is presented in Figure 10b. The image confirms the formation of spherical QDs with well-resolved lattice fringes and without distinct boundaries at the core-shell interface, which is due to only a minor mismatch (of merely 3%) between the PbSe and the PbS crystal structures. The corresponding FFT pattern shown in the inset confirms full crystallinity of the QDs. The XRD pattern (not shown here) is indicative of the rock-salt crystal structure, with the lattice parameter of ~6.12 Å. The PbSe/PbS QDs have the same structure as the original core PbSe QDs. The increase of the overall PbSe/PbS core/shell QD size by ~1.2 nm, as compared to the original PbSe cores, is consistent with one-ML PbS shell thickness. The preparation of PbSe/PbS core/shell QDs with the shell thickness >2 MLs required repeated (2–4 times) shell-precursor injections until the desired shell thickness was achieved. 

An HRTEM image of the PbSe/PbS core/shell QDs with *R*_S_*/R*_C_ = 3.0/1.5 nm is presented in Figure 10c, while an STM image of the PbSe/PbS core/shell QDs with *R*_S_*/R*_C_ = 4.0/1.5 nm is shown in Figure 10d. The STM image was obtained with a bias voltage of 2 V and a set-point current of 5 pA [114]. The inset presents a height profile of a single QD. The lateral dimensions of the profile are substantially larger than those of the real QD due to the tip width broadening, so only the height accurately indicates the QD size.

The synthesis of PbSe/PbSe*_x_*S_1–*x*_ core/alloyed-shell QDs is a one-step procedure [9]. The QDs were produced by simultaneous injection of Pb(OA)_2_, TOPSe, and TOPS into the reaction solution. The composition and thickness of the shell was controlled by adjusting the growth parameters such as monomer concentration, temperature, and reaction time. The formation of PbSe/PbSe*_x_*S_1–*x*_ (*x* > 0.1) QDs was achieved due to using the Se/S ratio >>1. An STEM image of the PbSe/PbSe*_x_*S_1–*x*_ core/alloyed-shell QDs with *R*_S_/*R*_C_ = 2.5/1.5 nm is shown in Figure 10e, an HRTEM image of a single QD being displayed in the inset. The TEM images show the formation of crystalline spherical QDs without any distinct boundary at the core-shell interface. The composition of the PbSe/PbSe*_x_*S_1–*x*_ QDs was determined by the XPS analysis (the data not shown here).

### 3.3. PbSe/CdSe Core/Shell Colloidal QDs with Band-Gap Energy in the Range of 0.8–1.1 eV

The synthesis of colloidal PbSe/CdSe QDs was performed in two stages. First, PbSe QDs were grown by the method described in Section 3.1. To exchange Pb^2+^ ions for Cd^2+^ ions on the QD surface, a batch of cadmium oleate (Cd(OA)_2_) was prepared by dissolving 5 mmols of CdO in 15 mmols of OA. The process took place under a nitrogen flow on a Shclenk line at 240 °C until a clear solution was obtained; then the solution was cooled to room temperature (RT). Subsequently, Cd(OA)_2_ solution was injected into the PbSe QDs dissolved in HDC, which initiated the cation-exchange process. To examine different Cd:Pb ratios, the concentration of PbSe was calculated using the previously published extinction coefficients and the sizing curve [109,110]. Various amounts of Cd(OA)_2_ were dissolved in HDC at temperatures ranging from 80 to 120 °C and mixed with PbSe QDs at different concentrations. Aliquots were taken for chemical and optical characterization after different time periods.

**Figure 11:**
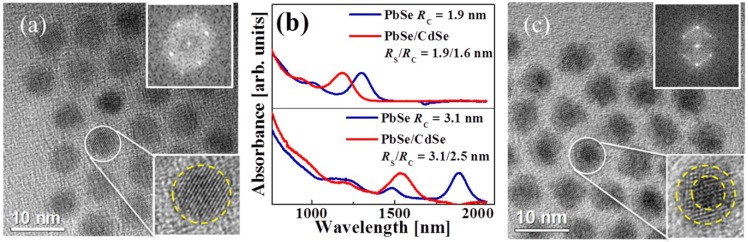
(**a**) HRTEM image of 6.2 ± 0.4 nm PbSe QDs. Insets: (bottom) an enlarged image of a single QD, (top) the corresponding FFT pattern; (**b**) absorption spectra of PbSe and PbSe/CdSe QDs with different *R*_S_*/R*_C_ ratios as indicated in the legend. Cation exchange reaction was performed at 100 °C for 30 min, Cd:Pb = 10 (bottom panel) and for 35 min, Cd:Pb = 50 (top panel); (**c**) HRTEM image of 6.2 ± 0.4 nm PbSe/CdSe. Insets: (bottom) an enlarged image of a single core/shell QD (*R*_S_*/R*_C_ = 3.1/2.5 nm), which shows clear contrast between the core and the shell components; (top) the corresponding FFT pattern. Cation-exchange reaction performed at 100 °C for 35 min, Cd:Pb = 50.

An HRTEM image of the original 6.2 ± 0.4 nm PbSe QDs is shown in Figure 11a. An enlarged HRTEM image of a single QD is displayed in the bottom inset. The images confirm the formation of spherical QDs with high crystallinity. The corresponding FFT pattern shown in the top inset is also indicative of full crystallinity of the QDs. The growth of a CdSe shell in the course of the above-mentioned cation-exchange reaction is accompanied by a pronounced blue shift of the PbSe/CdSe QD absorption spectra as compared to the original PbSe QD spectrum. Figure 11b shows absorption spectra of PbSe/CdSe QDs at various *R*_S_*/R*_C_ values (indicated in the figure). The results shown in the bottom and in the top panels were obtained with the QDs which were synthesized at 100 °C, the cation-exchange reaction lasting for 30 min (Cd:Pb = 10) and for 35 min (Cd:Pb = 50), respectively. The top panel in Figure 11b shows the absorption spectra of smaller original PbSe QDs (*R*_C_ = 1.9 nm) and of the corresponding PbSe/CdSe QDs with a thin shell (*R*_S_*/R*_C_ = 1.9/1.6 nm), while the bottom panel shows the absorption spectra of larger original PbSe QDs (*R*_C_ = 3.1 nm) and of the corresponding PbSe/CdSe QDs with a thin shell (*R*_S_*/R*_C_ = 3.1/2.5 nm). The thickness of the CdSe shell was estimated by absorption measurements of the original PbSe and of the final core/shell PbSe/CdSe QDs [71,100].

A representative HRTEM image of PbSe/CdSe core/shell QDs is shown in Figure 11c, while an enlarged image of a single core/shell QD with *R*_S_*/R*_C_ = 3.1/2.5 nm is presented in the bottom inset. The PbSe QDs shown in Figure 10a were used for the cation-exchange reaction, performed at 100 °C for 30 min. The image of a circled QD (Figure 11c) shows clear contrast between the core and the shell materials, without any distinct boundaries at the core/shell interface. It can be seen that the spherical form of the PbSe core remains unchanged and lattice fringes are well-resolved in the whole core/shell structure. The FFT pattern shown in the top inset of Figure 11c also confirms full crystallinity of the produced PbSe/CdSe core/shell QDs.

**Figure 12:**
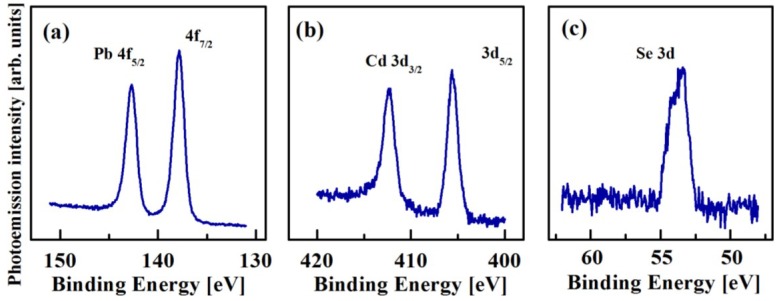
XPS spectra of air-free PbSe/CdSe QDs with *R*_S_*/R*_C_ = 2.3/2 nm. (**a**) Pb 4f peak; (**b**) Cd 3d peak; (**c**) Se 3d peak.

Representative XPS spectra of the air-free PbSe/CdSe QDs with *R*_S_*/R*_C_ = 3.1/2.5 nm are shown in Figure 12. The elemental analysis of different samples consistently showed the presence of all three elements: Pb, Cd, and Se, the characteristic Pb 4f, Cd 3d, and Se 3d XPS peaks, being displayed in Figure 12a–c. The 4f_5/2_ and 4f_7/2_ spin-orbit splitting of the Pb 4f level occurs at BE values of 142.8 and 138 eV, respectively, while the 3d_3/2_ and 3d_5/2_ spin-orbit splitting of the Cd 3d level occurs at BE values of 412.3 and 405.5 eV, respectively. As expected, no Cd signal was detected in PbSe QDs. The Se 3d_5/2_ XPS peak appears at the BE value of 54.1 eV. The characteristic BE values for Pb and Se do not differ from those obtained for PbSe (Figure 9 and Table 1).

## 4. Optical Properties of PbSe/PbSe*_x_*S_1–*x*_ (0 ≤ *x* ≤1) and PbSe/CdSe QD Heterostructures

A thorough investigation of the optical properties was performed by recording continuous-wave photoluminescence (cw-PL) spectra in oxygen-free environment at RT is described in Section 4.1. The change of the QD optical properties with temperature is described in Section 4.2.

### 4.1. Continuous-Wave Room-Temperature Photoluminescence Measurements in PbSe/PbSe_x_S_1–x_ and PbSe/CdSe QDs

**Figure 13:**
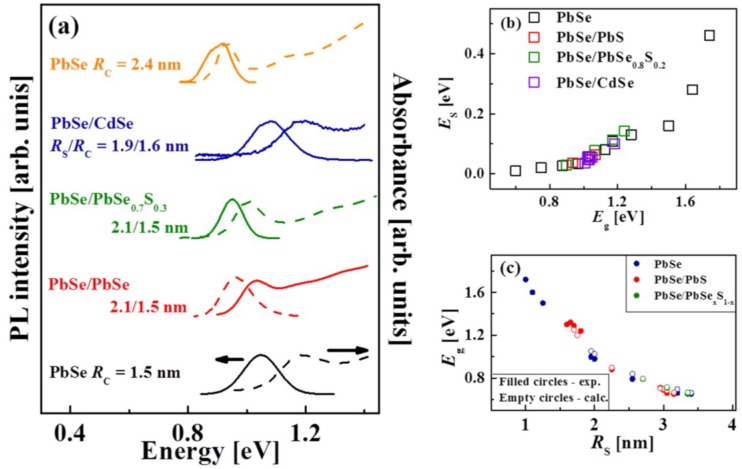
(**a**) Absorption (dashed lines) and emission (solid lines) spectra of PbSe core QDs and core/shell QD of various size, *R*s*/R*c ratio, and composition (see the legend); (**b**) dependence of Stokes-shift energy, *E*_S_, on *E_g_* for QDs of different composition (see the legend); (**c**) calculated (empty circles) and experimental (filled circles) values of the band-gap energy, *E*_g_, as a function of *R*s for various QDs (see the legend).

In what follows, the optical properties of various-sized core and core/shell QDs with the *E*_g_ of 1.2–0.92 eV are compared. Representative absorption and cw-PL spectra of a few QDs are shown in Figure 13a. The cw-PL spectra were obtained by means of non-resonant excitation at 2.3 eV. The bottom and top curves in Figure 13a correspond to PbSe core QDs with average radii *R*_C_ of 1.5 ± 0.2 and 2.4 ± 0.2 nm, respectively. The curves in the middle are the spectra of different QD heterostructures (PbSe/PbS and PbSe/CdSe core/shell QDs and PbSe/PbSe_0.7_S_0.3_ core/alloyed-shell QDs), their sizes being indicated in the figure. The absorption and PL curves of PbSe*_x_*S_1–*x*_ QDs are red-shifted as compared to the 1.5 nm PbSe cores, but they are blue-shifted as compared to the 2.4 nm PbSe cores. This midway shift is related to the quantum size effect combined with compositional tuning of *E*_g_. In contrast to this, the absorption and PL peaks in PbSe/CdSe core/shell heterostructures are blue-shifted as compared to the original PbSe QDs and correspond to the PbSe core lowest exciton transition, 1S_e_–1S_h_. The results presented in Figure 13a demonstrate that the 1S_e_–1S_h_ transition energy in 3.8 nm PbSe/CdSe QDs is higher than that in PbSe/PbSe*_x_*S_1–*x*_ QDs. This observation indicates that tuning of *E*_g_ can be achieved by changing not only the total QD size, but also the heterostructure composition, in accordance with the theoretical predictions discussed in Section 2.

Each cw-PL spectrum in Figure 13a shows an asymmetric PL band, its point of maximum being Stokes shifted (*E*_S_) with respect to the corresponding first-exciton absorption band. Figure 13b displays a plot of *E*_S_* vs.* *E*_g_ for the QDs with a thin shell listed in the legend. The *E*_S_ in the QDs may be accounted for by an increase of the QD size, the variation of the valley-valley and/or electron-hole exchange interactions. The *E_S_* variation in alloyed II–VI [115] and III–V QDs [116] was reported before and explained as being a specific effect of optical bowing [117] in those materials. 

The values of *E*_g_ obtained experimentally (filled circles) on the basis of the first-exciton absorption band and the *E*_g_ values calculated (empty circles) with regard for the Coulomb interaction corrections (discussed in Section 2) are presented in Figure 13c. It can be seen that the theoretical and experimental results are in good qualitative agreement. Since the *E*_g_ value in the PbSe/CdSe core/shell QDs depends only on the PbSe core size, the *E*_g_ dependence on the total QD size is not included in this figure.

**Figure 14:**
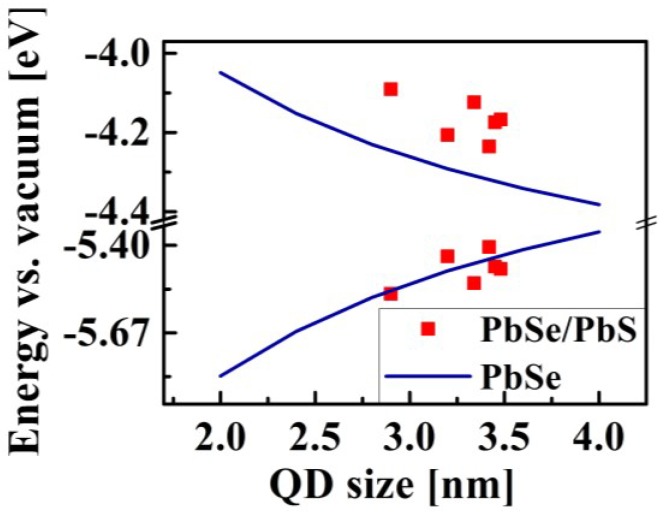
Absolute positions of the lowest quantized levels in PbSe and PbSe/PbS core/shell QDs of various size.

The absolute positions of the lowest quantized electron and hole energy levels in QDs, which are essential for their application in photovoltaic devices, are presented in Figure 14. These energy level values in the PbSe core (blue lines) and PbSe/PbS core/shell QDs (red dots), were estimated using the following formulas: E_e_ (d) = χ_bulk_ + ηE_conf._ (d) for electrons and E_h_ (d) = χ_bulk_ − *E*_g bulk_ − (1 − η) E_conf._ (d) for holes, where d is the QD diameter, χ_bulk_ is the bulk PbSe electron affinity, E_conf._ (d) = *E*_g QD_ (d) − *E*_g__bulk_ is the total confinement energy, and η is the fraction of E_conf._ acquired by the electron. The values of χ_bulk_ were taken from the cyclic voltammetry measurements reported before [118], the blue line was evaluated using the *E*_g__QD_ values from the empirical sizing curve [109], while the red dots were based on the experimental data obtained in this work (some of which are presented in Figure 14). According to the four-band **k•p** model, in the PbSe/PbS QDs studied here, the total confinement energy is distributed evenly between the electrons and the holes (which means that η = 0.5), while in the PbSe QDs, η = 0.42. It can be seen from Figure 14 that the conduction-edge energy in the PbSe/PbS QDs is higher than that in the PbSe QDs of the same size, which results in the heterostructure with altered band-gap energy relative to the vacuum level. These results correlate well with our previous calculations [95], and thus it can be suggested that the absolute energy of the band edges can be tuned with respect to the energy of the electrodes in various optoelectronic applications, thereby improving the performance efficiency.

**Figure 15:**
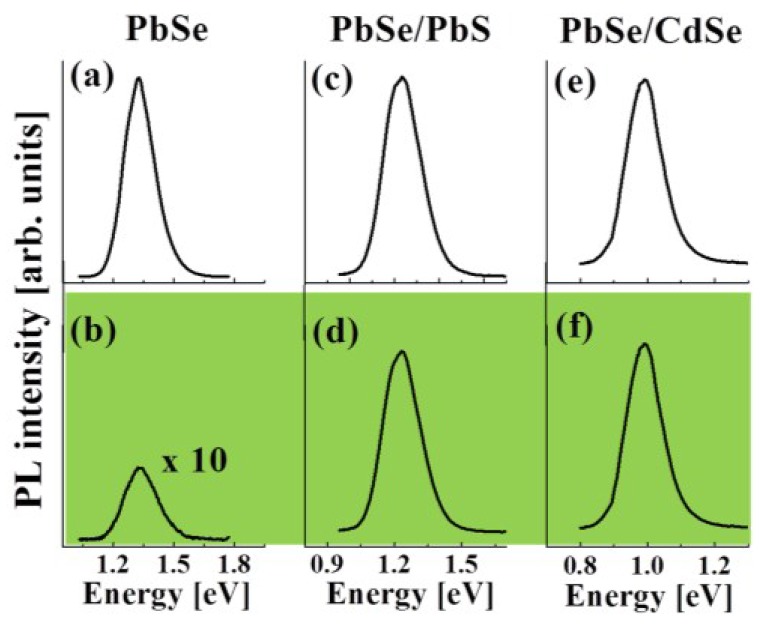
Comparison of the cw-PL spectra of PbSe (*R*_C_ = 1.1 nm), PbSe/PbS (*R*_S_*/R*_C_ = 1.6/1.1 nm), and PbSe/CdSe (*R*_S_*/R*_C_ = 1.9/1.6 nm) QDs recorded at RT under air-free conditions (**a**, **c**, and **e**, respectively) and after exposure to air for 20 min (**b**, **d**, and **f**, respectively).

Regarding the potential application of colloidal QDs in various optoelectronic devices, the problem of the QD chemical and photochemical stability is of major concern. For this reason, we investigated the stability of the QDs obtained here by following the changes of the PbSe, PbSe/PbS, and PbSe/CdSe core/shell QD PL spectra measured after exposure to air for a controlled period of time. Representative cw-PL spectra of PbSe QDs (*R*_C_ = 1.1 nm), PbSe/PbS (*R*_S_*/R*_C_ = 1.6/1.1 nm), and PbSe/CdSe (*R*_S_*/R*_C_ = 1.9/1.6 nm) core/shell QDs are shown in Figure 14. The spectra were recorded at RT under air-free conditions (Figure 15a,c,e) as well as after 20 min of air-exposure (Figure 15b,d,f). From the curves presented in Figure 15, it can be seen that air-exposure leads to a 30-fold reduction of the PbSe QD PL intensity and to the peak blue-shift by 20 meV, while the PL intensity and energy in the thin-shell PbSe/PbS and PbSe/CdSe core/shell QDs remain nearly unchanged. The air-induced emission quenching in PbSe QDs is presumably associated with the formation of trapping sites due to surface oxidation [55,119]. According to our results, the formation of a thin PbS (~0.5 nm) or CdSe (~0.3 nm) shell is sufficient for protecting the core QDs from immediate oxidation.

### 4.2. Thermally Activated Processes in PbSe/PbSe_x_S_1–x_ and PbSe/CdSe QDs

**Figure 16:**
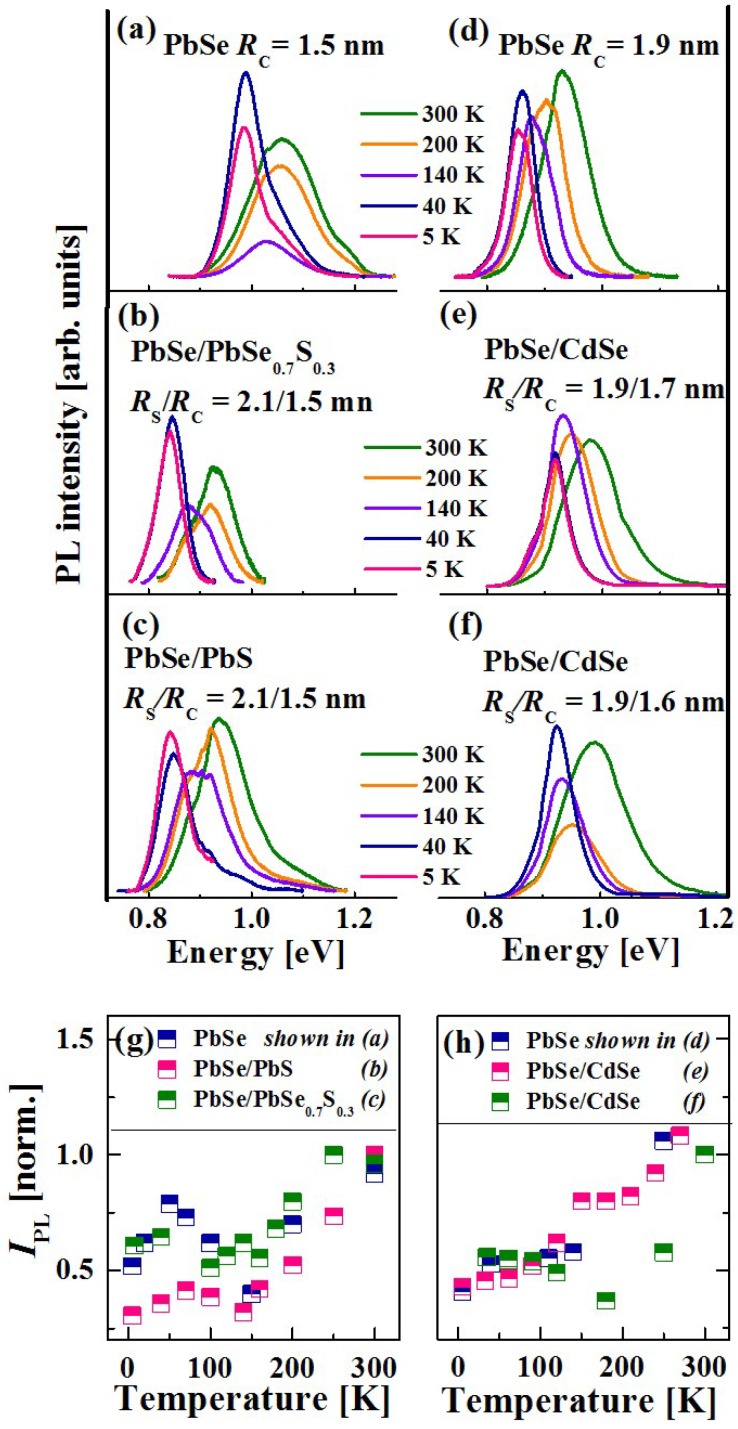
cw-PL spectra of air-free (**a**), (**d**) PbSe core; (**b**) PbSe/PbS core/shell; (**c**) PbSe/PbSe_0.7_S_0.3_ core/alloyed-shell; (**e**,**f**) PbSe/CdSe core/shell QDs with different *Rs/Rc* values (see the legend); (**g**,**h**) plot of normalized cw-PL integrated intensity, *I_PL_*,* vs.* temperature for the same QDs as in (**a**–**f**), see the legend.

For temperature-dependent measurements the QDs were dispersed in 2,2,4,4,6,8,8-heptamethylnonane, a glass-forming solution, and excited at 2.3 eV. Figure 16a–f display a set of cw-PL spectra of air-free QDs with a similar core size. The spectra of PbSe core QDs (a and d), and of the corresponding PbSe/PbSe_0.7_S_0.3_ (b), PbSe/PbS (c), and PbSe/CdSe (e and f) core/shell QDs were recorded at various temperatures ranging from 5 to 300 K.

The cw-PL spectra exhibit a red shift with the temperature decrease, resembling the trend found in low-energy band-gap semiconductors [120]. Moreover, most of the PL curves possess an asymmetric emission band, presumably arising from two overlapping recombination events. The split energy varies from 55 meV to 80 meV, the former and the latter values corresponding to the largest and the smallest QDs studied, respectively. It should be noted that the split energy remains almost constant with the temperature increase although the high-energy component becomes the dominant band at elevated temperatures. It was found that the relative intensity of the two emission bands is almost the same. 

The nature of the emission bands was examined by varying the pumping intensity. The occurrence of multiple-exciton emission under strong pumping conditions was excluded due to the long lifetime of the unresolved emission band (see below). The possibility of trapped-carrier recombination was also excluded due to the lack of a saturation effect at the highest pumping power. More likely, the entire emission spectrum is related to band-edge recombination. It should be mentioned that an asymmetric band or even two distinct emission bands in non-resonant cw-PL spectra of colloidal IV–VI QDs were already observed in previous studies [2,56,90,121,122]. Presumably, this phenomenon is accounted for by inter-valley coupling at the L-points of the Brillouin zone [8,84,121]. Furthermore, the study of binary PbSe QDs by the fluorescence line narrowing method revealed Stokes and anti-Stokes shifts, associated with the existence of two bands [123,124]. The existence of two bands may be related to degeneracy splitting of the band-edge states due to a slight shape anisotropy of the QDs [108].

The dependence of normalized integrated PL intensity (*I_PL_*) on temperature (*T*) for air-free PbSe-based QDs is shown in Figure 16g,h. The values of *I_PL_*(T) were normalized to the *I_PL_* value obtained at RT. The plots are characterized by a mild increase of *I_PL_*(T) between 5 K and 50 K, which correlates with a phonon-assisted transition from a dark-to-bright exciton emission [125], followed by emisssion quenching, which may be associated with carrier trapping. Eventually, at the temperatures above 150 K, *I_PL_* increases and reaches its maximal value at RT. The maximal intensity at RT corresponds to the recombination from a bright-exciton state [125], which is, presumably, enhanced by phonon-assisted de-trapping from shallow non-radiative defect states. It can be seen that the plot of *I_PL_*(T) in PbSe/CdSe (Figure 16h) QDs follows the same trend as PbSe and PbSe/PbS QDs.

**Figure 17:**
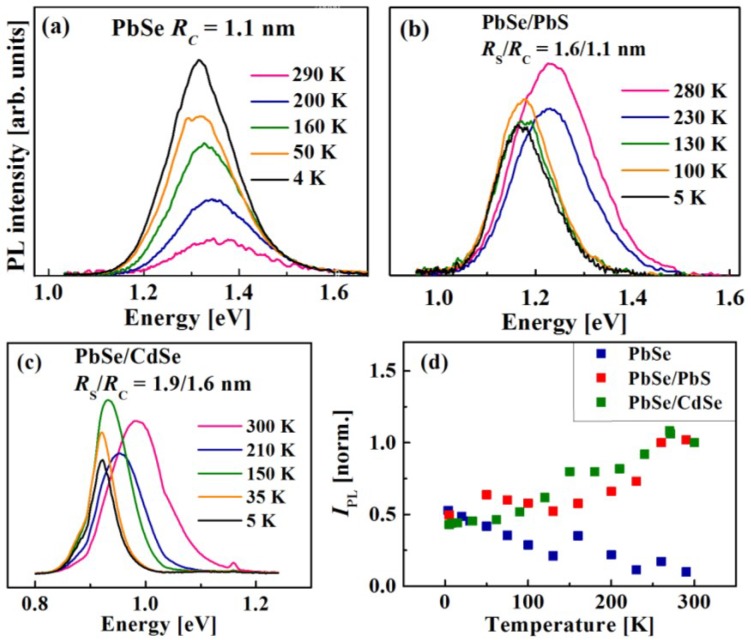
cw-PL spectra of QDs exposed to air for 20 min. (**a**) PbSe; (**b**) PbSe/PbS, and (**c**) PbSe/CdSe QDs with different *R*_S_*/R*_C_ values (see the legends); (**d**) dependence of* I_PL_* on temperature for the same QDs as in (**a**–**c**), exposed to air for 20 and 50 min.

A set of measurements performed to further study the behavior of IV–VI QDs on exposure to air gave results presented in Figure 17. The cw-PL spectra of PbSe core QDs (Figure 17a), recorded at different temperatures, illustrate the luminescence intensity reduction with the temperature increase after 20 min of air exposure. The observed drastic drop of the PL intensity may be associated with thermally-activated trapping into surface defect states, produced by QD oxidation [96]. Figure 17b,c shows the cw-PL curves for PbSe/PbS (*R*_S_*/R*_C_ = 1.6/1.1 nm) and PbSe/CdSe (*R*_S_/*R*_C_ = 1.9/1.6 nm) QDs recorded in a similar temperature range and with the same time of air-exposure as the curves for PbSe QDs in Figure 17a. It can be seen that the exposure of core/shell QDs to ambient conditions did not result in quenching their luminescence intensity. On the contrary, the *I_PL_* increase observed in air-free core/shell QDs at RT also occurs in this case. Figure 17d displays the plots of normalized *I_PL_ vs.* *T* for the same QDs as in Figure 17b,c, but after air-exposure for various periods of time (from 20 to 50 min). The plots indicate that air-exposure for limited time does not result in dramatic luminescence changes, while after a certain period of time the QDs may be oxidized, with subsequent quenching of the luminescence intensity [56,96]. Although the data suggest that the core/shell QDs remain chemically stable only during a restricted time of air-exposure, it can be crucially beneficial for certain processes involved in the fabrication of QD-based devices.

### 4.3. Temperature Dependent Time-Resolved Photoluminescence of PbSe/PbSe_x_S_1–x_ (0 ≤ x ≤ 1) and PbSe/CdSe QDs

**Figure 18:**
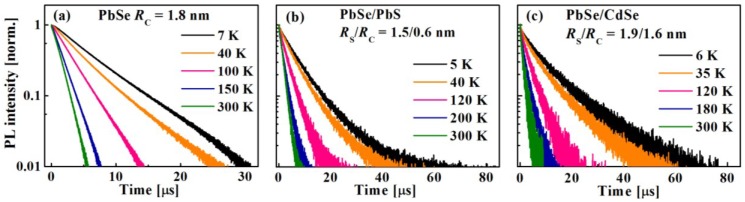
Representative time-resolved PL decay curves recorded at various temperatures for (**a**) PbSe; (**b**) PbSe/PbS; and (**c**) PbSe/CdSe QDs with different *R*s*/R*c values as indicated in the legends.

The PL intensity decay curves were obtained by exciting the sample at 2.4 eV and following the intensity variation over time. The normalized PL decay curves for PbSe (*R_C_* = 1.8 nm) core QDs and for PbSe/PbS (*R*_S_*/R*_C_ = 1.5/0.6 nm), and PbSe/CdSe (*R*_S_*/R*_C_ = 1.9/1.6 nm) core/shell QDs shown in Figure 18a,b,c, respectively, were recorded under air-free conditions at various temperatures. 

**Figure 19:**
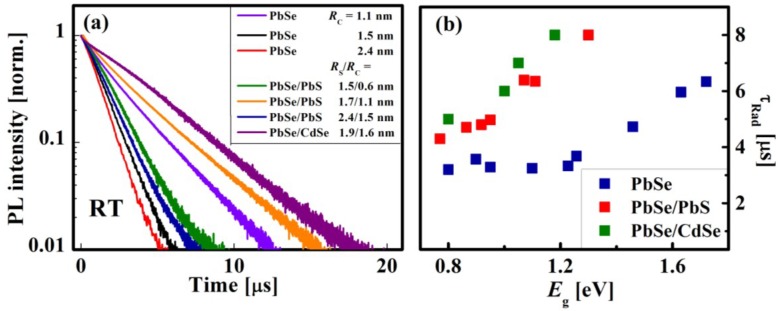
(**a**) Representative RT PL decay curves for QDs of different composition, *R*_C_, and *R*_S_*/R*_C_ ratios (see the legend); (**b**) dependence of radiative life-time, τ_Rad_, on the size and composition of the QDs listed in the legend.

The curves are best fitted either to a single exponent, *I*(*t*) = *A*exp(−*t*/τ_0_), or to a double exponent, *I*(*t*) = *A*_1_exp(−*t*/τ_1_) + *A*_2_exp(−*t*/τ_2_), with the weighted decay time

(3) $${\text{τ}}_{0}=\frac{A_{1}^{2}{\text{τ}}_{1}^{2}+A_{2}^{2}{\text{τ}}_{2}^{2}}{A_{1}{\text{τ}}_{1}+A_{2}{\text{τ}}_{2}}$$

where *A* is a pre-exponential factor and τ_0_ is the measured exciton lifetime. Figure 19a shows the RT PL decay curves of PbSe, PbSe/PbS, and PbSe/CdSe QDs,* R*_C_ and the *R*_S_*/R*_C_ ratios being indicated in the legend. As a rule, the value of τ_0_ decreases from 4.2 µs to 1.4 µs with the growth of the core radius from 1.1 to 2.4 nm and with the variation of the core-to-shell division [55,126,127]. At a fixed temperature, the value of τ_0_ depends on the radiative (τ_Rad_) and nonradiative (τ_NRad_) processes, according to the following relation [128,129]:

(4) $$\frac{1}{{\text{τ}}_{0}}=\frac{1}{{\text{τ}}_{\text{rad}}}+\frac{1}{{\text{τ}}_{\text{nrad}}}$$

The values of τ_Rad_ are appropriate to consider for comparing the behavior of core/shell and core QDs or for the comparison with the literature data [55,85]. The plots of τ_Rad_* vs.* *E_g_* for PbSe, PbSe/PbS, and PbSe/CdSe QDs, measured at RT are presented in Figure 19b. It can be seen that the values of τ_Rad_ are larger in the core/shell QDs as compared to the corresponding cores [84]. This effect can be explained by the large dielectric constant of PbSe [85] and by mixing of adjacent electronic-band minima [84]. It should be emphasized that such a long exciton lifetime in the core/shell QDs is beneficial for charge extraction in QD-based PV devices.

## 5. Applications

PbSe, PbSe/PbS core/shell, and PbSe/PbSe*_x_*S_1–*x*_ core/alloyed-shell QD heterostructures with a band gap of 0.8 eV, dispersed in a PMMA polymer, were used in the eye-safe Erbium glass laser. It was found that the QDs act as fast saturable absorbers, with effective lifetime τ_eff_ ≈ 4.0 ps and a relatively large ground-state cross-section of absorption (σ_gs_ ≈ 10^−15^ cm^2^). The τ_eff_ value is accounted for by the formation of multi-excitons at the used pumping power. The product of σ_gs_ and τ_eff_ ensures sufficient Q-switching performance and tunability in the NIR spectral range. The laser properties could be improved by using PbSe/PbSe*_x_*S_1–*x*_ core/alloyed-shell QD heterostructures [18,19,123].

Moreover, QD-based gain devices are characterized by amplified spontaneous emission (ASE) under conditions suitable for technological applications such as optical pumping by a continuous diode laser under RT conditions. The optical gain and the ASE properties were determined with a 4.2 µm-thick QDs-doped PMMA film (arranged on a silicon wafer), with a variable (0.01–0.2 cm) stripe length and pump intensity, using a cw laser diode at 980 nm as a pump source. The pump beam was concentrated by a cylindrical lens and a narrow strip of uniform intensity was selected using a 300 µm-wide slit made of aluminum foil directly deposited on the film surface. Of course, the ASE band was narrower than the spontaneous emission band, but the width of the former was independent of the pumping power, which presumably suggested the absence of inhomogeneous broadening due to the QD-size distribution. The results revealed a relatively large gain parameter g = 2.63–6.67 cm^−1^ [123].

Furthermore, the construction of solid-state high-efficiency QDs/TiO_2_ heterojunction solar cells, in which small-sized PbSe/PbS core/shell QD heterostructures with a band gap in the range of 1.1–1.3 eV were used, allowed achieving improved parameters such as power conversion efficiency of 4.5% with short-circuit photocurrent (*J*sc) of 17.3 mA/cm^2^. The PbS shell covering the PbSe core should offer sufficient protection from fast oxygen penetration, providing the PbSe/PbS QD heterostructures with low surface trapping and chemical and photo-chemical stability, which is essential for successful integration of QDs into solar-cell devices [17].

The PbSe-based QD heterostructures could appear highly beneficial in the QD applications where the material size is restricted, e.g., in biology or in various opto-electronic devices, which require closely packed self-assemblies of relatively small QDs. However, all applications impose stringent requirements on the optical tunability of the materials. We have shown that the problem can be resolved by designing core/(alloyed)-shell heterostructures, where the charge carrier confinement can be tuned by changing the heterostructure configuration.

## 6. Future Outlook

Although Pb-chalcogenide-based core/shell structures are being widely investigated, there are still many challenges that have to be met. Small band-gap energy values of lead chalcogenides make them good candidates for QD-based solar cells, which utilize multiple exciton generation (MEG). QD core/shell structures may provide an efficient way for surface passivation and suppression of competing processes, including: Auger relaxation, as well as energy transfer to surface ligands or to surface states [130]. Using (quasi-)type-II QDs with thick shells can improve MEG due to reduced carrier overlapping [131]. However, as already mentioned above, a thick shell layer limits the charge extraction efficiency and reduces the current density [132]. This problem can be overcome by providing free charge carriers through controlled doping of the QD or the shell layer [133]. The doping procedure can also be used to manipulate charge transfer rates [134] due to its effect on the Fermi level [134]. Optimization of the shell composition and architecture is a promising avenue towards the MEG implementation. 

Such methods as* in vivo* fluorescence optical molecular imaging and photothermal therapy are quickly emerging fields of medicine. Probing and exciting certain sites located deep in biological tissues requires high-QY emitters, operating in the NIR spectral region [135], which is characteristic of the Pb-chalcogenide emission spectrum. However, the emitter surface has to be properly treated to make it biocompatible. In view of the fact that direct application of silica coating or water-soluble ligands quenches the emission [136], more complicated core/shell heterostructures (e.g., PbSeCdSeZnS) might be required. Such structures can be produced in the course of a cation-exchange process [71], the problem often encountered being only partial coating obtained in this way. Although the technique is being widely used, the nature of the mechanisms involved is still unclear [136]. Therefore, investigation of the cation-exchange mechanism in PbSe-based QDs is essential for their successful biological and medical application.

Furthermore, any future implementation of core/shell QDs in new-generation electronic devices will require their prolonged exposure to air and to microelectronics fabrication processes. Thus, additional further research is required into the core/shell QD stability under the above-mentioned conditions.

## 7. Summary

Colloidal core/shell heterostructures, such as PbSe/PbSe*_x_*S_1–*x*_ (0 ≤ *x* ≤ 1) and thin-shell PbSe/CdSe QDs, were described in this review article. These QDs demonstrate tunable band-gap energy and high crystallographic and dielectric match between the core and the shell constituents. Moreover, the overall electronic properties and carrier-distribution functions can be controlled by the core/shell architecture. Besides, these heterostructures show chemical and photochemical stability. The electronic band structure in PbSe/PbS QDs was evaluated in the framework of the **k•p** model. The model predicts a wide range of physical properties on the basis of effective mass anisotropy and different dielectric constants of QD constituents. The model shows the possibility of constructing heterostructures with desired composition and optical properties. PbSe/CdSe QDs were modeled by single band effective mass model in FEA software (COMSOL). The model shows different confinement regimes for the electron and hole and their relevant radial distribution. This approach is useful for evaluation of charge carriers’ behavior in complicate geometric structures that cannot be treated analytically. A thorough investigation of the QD optical properties was performed by recording continuous-wave and time-resolved PL spectra at various temperatures. The energy shifts and band-edge temperature stability were assessed by alleviating dark-bright splitting induced by exchange and/or valley-valley interactions, and by measuring the radiative lifetime. The results were compared to the currently known properties of PbSe QDs. The results suggest the uniqueness of the QD electronic properties, which can be controlled by the shell thickness and the alloyed composition of these heterostructures.

## References

[1] Kang I., Wise F.W. (1997). Electronic structure and optical properties of PbS and PbSe quantum dots. OSA J. Opt. Soc. Am. B.

[2] Du H., Chen C., Krishnan R., Krauss T.D., Harbold J.M., Wise F.W., Thomas M.G., Silcox J. (2002). Optical properties of colloidal PbSe nanocrystals. Nano Lett..

[3] Wehrenberg B.L., Wang C., Guyot-Sionnest P. (2002). Interband and intraband optical studies of PbSe colloidal quantum dots. J. Phys. Chem. B.

[4] Talapin D.V., Murray C.B. (2005). Pbse nanocrystal solids for n- and p-channel thin film field-effect transistors. Science.

[5] Madelung O. (2004). Semiconductors: Data Handbook.

[6] Romero H.E., Drndic M. (2005). Coulomb blockade and hopping conduction in PbSe quantum dots. Phys. Rev. Lett..

[7] Wise F.W. (2000). Lead salt quantum dots: The limit of strong quantum confinement. Acc. Chem. Res..

[8] Harbold J.M., Du H., Krauss T.D., Cho K.-S., Murray C.B., Wise F.W. (2005). Time-resolved intraband relaxation of strongly confined electrons and holes in colloidal PbSe nanocrystals. Phys. Rev. B.

[9] Brumer M., Kigel A., Amirav L., Sashchiuk A., Solomesch O., Tessler N., Lifshitz E. (2005). PbSe/PbS and PbSe/PbSe_x_S_1–x_ core/shell nanocrystals. Adv. Funct. Mater..

[10] Pietryga J.M., Schaller R.D., Werder D., Stewart M.H., Klimov V.I., Hollingsworth J.A. (2004). Pushing the band gap envelope: Mid-infrared emitting colloidal PbSe quantum dots. J. Am. Chem. Soc..

[11] Nozik A.J. (2010). Nanoscience and nanostructures for photovoltaics and solar fuels. Nano Lett..

[12] Nozik A.J. (2002). Quantum dot solar cells. Phys. E Low Dimens. Syst. Nanostructures.

[13] Tang J., Kemp K.W., Hoogland S., Jeong K.S., Liu H., Levina L., Furukawa M., Wang X., Debnath R., Cha D. (2011). Colloidal-quantum-dot photovoltaics using atomic-ligand passivation. Nat. Mater..

[14] Ip A.H., Thon S.M., Hoogland S., Voznyy O., Zhitomirsky D., Debnath R., Levina L., Rollny L.R., Carey G.H., Fischer A. (2012). Hybrid passivated colloidal quantum dot solids. Nat. Nano.

[15] Ma W., Swisher S.L., Ewers T., Engel J., Ferry V.E., Atwater H.A., Alivisatos A.P. (2011). Photovoltaic performance of ultrasmall PbSe quantum dots. ACS Nano.

[16] Barkhouse D.A.R., Debnath R., Kramer I.J., Zhitomirsky D., Pattantyus-Abraham A.G., Levina L., Etgar L., Grätzel M., Sargent E.H. (2011). Depleted bulk heterojunction colloidal quantum dot photovoltaics. Adv. Mater..

[17] Etgar L., Yanover D., Čapek R.K., Vaxenburg R., Xue Z., Liu B., Nazeeruddin M.K., Lifshitz E., Grätzel M. (2013). Core/shell PbSe/PbS qds tio 2 heterojunction solar cell. Adv. Functual.

[18] Brumer M., Sirota M., Kigel A., Sashchiuk A., Galun E., Burshtein Z., Lifshitz E. (2006). Nanocrystals of PbSe core, PbSe/PbS, and PbSe/PbSe_x_S_1–x_ core/shell as saturable absorbers in passively q-switched near-infrared lasers. Appl. Opt..

[19] Sirota M., Galun E., Krupkin V., Glushko A., Kigel A., Brumer M., Sashchiuk A., Amirav L., Lifshitz E., Andrews D.L., Cao G.Z., Gaburro Z. (2004). Nanophotonic materials. IV–VI Semiconductor Nanocrystals for Passive Q-switch in IR.

[20] Wang R.Y., Feser J.P., Lee J.-S., Talapin D.V., Segalman R., Majumdar A. (2008). Enhanced thermopower in PbSe nanocrystal quantum dot superlattices. Nano Lett..

[21] Petta J.R., Johnson A.C., Taylor J.M., Laird E.A., Yacoby A., Lukin M.D., Marcus C.M., Hanson M.P., Gossard A.C. (2005). Coherent manipulation of coupled electron spins in semiconductor quantum dots. Science.

[22] Loss D., DiVincenzo D.P. (1998). Quantum computation with quantum dots. Phys. Rev. A.

[23] Alivisatos P. (2004). The use of nanocrystals in biological detection. Nat. Biotechnol..

[24] Dubertret B. (2005). Quantum dots: Dna detectives. Nat. Mater..

[25] Smith A.M., Nie S. (2009). Next-generation quantum dots. Nat. Biotechnol..

[26] Tischler J.G., Kennedy T.A., Glaser E.R., Efros A.L., Foos E.E., Boercker J.E., Zega T.J., Stroud R.M., Erwin S.C. (2010). Band-edge excitons in PbSe nanocrystals and nanorods. Phys. Rev. B.

[27] Rubin-Brusilovski A., Maikov G., Kolan D., Vaxenburg R., Tilchin J., Kauffmann Y., Sashchiuk A., Lifshtiz E. (2012). Influence of alloying on the optical properties of IV–VI nanorods. J. Phys. Chem. C.

[28] Casavola M., van Huis M.A., Bals S., Lambert K., Hens Z., Vanmaekelbergh D. (2011). Anisotropic cation exchange in PbSe/CdSe core/shell nanocrystals of different geometry. Chem. Mater..

[29] Boercker J.E., Clifton E.M., Tischler J.G., Foos E.E., Zega T.J., Twigg M.E., Stroud R.M. (2011). Size and temperature dependence of band-edge excitons in PbSe nanowires. J. Phys. Chem. Lett..

[30] Sashchiuk A., Amirav L., Bashouti M., Krueger M., Sivan U., Lifshitz E. (2003). Pbse nanocrystal assemblies: Synthesis and structural, optical, and electrical characterization. Nano Lett..

[31] Mokari T., Habas S.E., Zhang M., Yang P. (2008). Synthesis of lead chalcogenide alloy and core/shell nanowires. Angew. Chem. Int. Ed..

[32] Bashouti M., Lifshitz E. (2007). Pbs sub-micrometer structures with anisotropic shape: Ribbons, wires, octapods, and hollowed cubes. Inorg. Chem..

[33] Lifshitz E., Bashouti M., Kloper V., Kigel A., Eisen M.S., Berger S. (2003). Synthesis and characterization of PbSe quantum wires, multipods, quantum rods, and cubes. Nano Lett..

[34] Schliehe C., Juarez B.H., Pelletier M., Jander S., Greshnykh D., Nagel M., Meyer A., Foerster S., Kornowski A., Klinke C. (2010). Ultrathin PbS sheets by two-dimensional oriented attachment. Science.

[35] Cho K.-S., Talapin D.V., Gaschler W., Murray C.B. (2005). Designing PbSe nanowires and nanorings through oriented attachment of nanoparticles. J. Am. Chem. Soc..

[36] Koh W., Bartnik A.C., Wise F.W., Murray C.B. (2010). Synthesis of monodisperse PbSe nanorods: A case for oriented attachment. J. Am. Chem. Soc..

[37] Yang C., Zhou X., Wang L., Tian X., Wang Y., Pi Z. (2009). Preparation and tunable photoluminescence of alloyed cdSxse_1−x_ nanorods. J. Mater. Sci..

[38] Bealing C.R., Baumgardner W.J., Choi J.J., Hanrath T., Hennig R.G. (2012). Predicting nanocrystal shape through consideration of surface-ligand interactions. ACS Nano.

[39] Lobo A., Borchert H., Talapin D.V., Weller H., Möller T. (2006). Surface oxidation of CdTe nanocrystals—A high resolution core-level photoelectron spectroscopy study. Colloids Surf. A Physicochem. Eng. Asp..

[40] Efros A.L., Rosen M. (2000). The electronic structure of semiconductor nanocrystals. Annu. Rev. Mater. Sci..

[41] Nirmal M., Norris D.J., Kuno M., Bawendi M.G., Efros A.L., Rosen M. (1995). Observation of the “Dark exciton” In CdSe quantum dots. Phys. Rev. Lett..

[42] Kigel A., Brumer M., Maikov G., Sashchiuk A., Lifshitz E. (2009). The ground-state exciton lifetime of PbSe nanocrystal quantum dots. Superlattices Microstruct..

[43] Nozik A.J., Beard M.C., Luther J.M., Law M., Ellingson R.J., Johnson J.C. (2010). Semiconductor quantum dots and quantum dot arrays and applications of multiple exciton generation to third-generation photovoltaic solar cells. Chem. Rev..

[44] Nair G., Geyer S.M., Chang L.-Y., Bawendi M.G. (2008). Carrier multiplication yields in PbS and PbSe nanocrystals measured by transient photoluminescence. Phys. Rev. B.

[45] Schaller R.D., Klimov V.I. (2004). High efficiency carrier multiplication in PbSe nanocrystals: Implications for solar energy conversion. Phys. Rev. Lett..

[46] Ellingson R.J., Beard M.C., Johnson J.C., Yu P., Micic O.I., Nozik A.J., Shabaev A., Efros A.L. (2005). Highly efficient multiple exciton generation in colloidal PbSe and PbS quantum dots. Nano Lett..

[47] Trinh M.T., Houtepen A.J., Schins J.M., Hanrath T., Piris J., Knulst W., Goossens A.P.L.M., Siebbeles L.D.A. (2008). In spite of recent doubts carrier multiplication does occur in PbSe nanocrystals. Nano Lett..

[48] McGuire J.A., Joo J., Pietryga J.M., Schaller R.D., Klimov V.I. (2008). New aspects of carrier multiplication in semiconductor nanocrystals. Acc. Chem. Res..

[49] Ji M., Park S., Connor S.T., Mokari T., Cui Y., Gaffney K.J. (2009). Efficient multiple exciton generation observed in colloidal PbSe quantum dots with temporally and spectrally resolved intraband excitation. Nano Lett..

[50] Beard M.C., Midgett A.G., Law M., Semonin O.E., Ellingson R.J., Nozik A.J. (2009). Variations in the quantum efficiency of multiple exciton generation for a series of chemically treated PbSe nanocrystal films. Nano Lett..

[51] Kim S.J., Kim W.J., Sahoo Y., Cartwright A.N., Prasad P.N. (2008). Multiple exciton generation and electrical extraction from a PbSe quantum dot photoconductor. Appl. Phys. Lett..

[52] Trinh M.T., Polak L., Schins J.M., Houtepen A.J., Vaxenburg R., Maikov G.I., Grinbom G., Midgett A.G., Luther J.M., Beard M.C. (2011). Anomalous independence of multiple exciton generation on different group IV−VI quantum dot architectures. Nano Lett..

[53] Cunningham P.D., Boercker J.E., Foos E.E., Lumb M.P., Smith A.R., Tischler J.G., Melinger J.S. (2011). Enhanced multiple exciton generation in quasi-one-dimensional semiconductors. Nano Lett..

[54] Murphy J.E., Beard M.C., Norman A.G., Ahrenkiel S.P., Johnson J.C., Yu P., Mićić O.I., Ellingson R.J., Nozik A.J. (2006). Pbte colloidal nanocrystals: Synthesis, characterization, and multiple exciton generation. J. Am. Chem. Soc..

[55] Sykora M., Koposov A.Y., McGuire J.A., Schulze R.K., Tretiak O., Pietryga J.M., Klimov V.I. (2010). Effect of air exposure on surface properties, electronic structure, and carrier relaxation in PbSe nanocrystals. ACS Nano.

[56] Chappell H.E., Hughes B.K., Beard M.C., Nozik A.J., Johnson J.C. (2011). Emission quenching in PbSe quantum dot arrays by short-term air exposure. J. Phys. Chem. Lett..

[57] Yanover D., Vaxenburg R., Tilchin J., Rubin-Brusilovski A., Zaiats G., Čapek R.K., Sashchiuk A., Lifshitz E. (2014). Significance of small-sized PbSe/PbS core/shell colloidal quantum dots for optoelectronic applications. J. Phys. Chem. C.

[58] Kovalenko M.V., Bodnarchuk M.I., Zaumseil J., Lee J.-S., Talapin D.V. (2010). Expanding the chemical versatility of colloidal nanocrystals capped with molecular metal chalcogenide ligands. J. Am. Chem. Soc..

[59] Bae W.K., Joo J., Padilha L.A., Won J., Lee D.C., Lin Q., Koh W., Luo H., Klimov V.I., Pietryga J.M. (2012). Highly effective surface passivation of PbSe quantum dots through reaction with molecular chlorine. J. Am. Chem. Soc..

[60] Hughes B.K., Ruddy D.A., Blackburn J.L., Smith D.K., Bergren M.R., Nozik A.J., Johnson J.C., Beard M.C. (2012). Control of PbSe quantum dot surface chemistry and photophysics using an alkylselenide ligand. ACS Nano.

[61] Gao M., Kirstein S., Möhwald H., Rogach A.L., Kornowski A., Eychmüller A., Weller H. (1998). Strongly photoluminescent CdTe nanocrystals by proper surface modification. J. Phys. Chem. B.

[62] Kloper V., Osovsky R., Kolny-Olesiak J., Sashchiuk A., Lifshitz E. (2007). The growth of colloidal cadmium telluride nanocrystal quantum dots in the presence of cd0 nanoparticles. J. Phys. Chem. C.

[63] Moreels I., Fritzinger B., Martins J.C., Hens Z. (2008). Surface chemistry of colloidal PbSe nanocrystals. J. Am. Chem. Soc..

[64] Abel K.A., FitzGerald P.A., Wang T.-Y., Regier T.Z., Raudsepp M., Ringer S.P., Warr G.G., van Veggel F.C.J.M. (2012). Probing the structure of colloidal core/shell quantum dots formed by cation exchange. J. Phys. Chem. C.

[65] Fritzinger B., Capek R.K., Lambert K., Martins J.C., Hens Z. (2010). Utilizing self-exchange to address the binding of carboxylic acid ligands to CdSe quantum dots. J. Am. Chem. Soc..

[66] Tracy J.B., Weiss D.N., Dinega D.P., Bawendi M.G. (2005). Exchange biasing and magnetic properties of partially and fully oxidized colloidal cobalt nanoparticles. Phys. Rev. B.

[67] Mukherjee B., Peterson A., Subramanian V.R. (2012). 1D CdS/PbS heterostructured nanowire synthesis using cation exchange. Chem. Commun..

[68] Smith A.M., Nie S. (2011). Bright and compact alloyed quantum dots with broadly tunable near-infrared absorption and fluorescence spectra through mercury cation exchange. J. Am. Chem. Soc.

[69] Lai L.-H., Protesescu L., Kovalenko M.V., Loi M.A. (2014). Sensitized solar cells with colloidal PbS-CdS core-shell quantum dots. Phys. Chem. Chem. Phys..

[70] Zhao H., Liang H., Vidal F., Rosei F., Vomiero A., Ma D. (2014). Size dependence of temperature-related optical properties of PbS and PbS/CdS core/shell quantum dots. J. Phys. Chem. C.

[71] Pietryga J.M., Werder D.J., Williams D.J., Casson J.L., Schaller R.D., Klimov V.I., Hollingsworth J.A. (2008). Utilizing the lability of lead selenide to produce heterostructured nanocrystals with bright, stable infrared emission. J. Am. Chem. Soc..

[72] Liu Y., Gibbs M., Perkins C.L., Tolentino J., Zarghami M.H., Bustamante J., Law M. (2011). Robust, functional nanocrystal solids by infilling with atomic layer deposition. Nano Lett..

[73] Ma W., Luther J.M., Zheng H., Wu Y., Alivisatos A.P. (2009). Photovoltaic devices employing ternary PbS_x_Se_1–x_ nanocrystals. Nano Lett..

[74] Sashchiuk A., Yanover D., Rubin-Brusilovski A., Maikov G.I., Capek R.K., Vaxenburg R., Tilchin J., Zaiats G., Lifshitz E. (2013). Tuning of electronic properties in IV–VI colloidal nanostructures by alloy composition and architecture. Nanoscale.

[75] Efros A.L., Rosen M., Kuno M., Nirmal M., Norris D.J., Bawendi M. (1996). Band-edge exciton in quantum dots of semiconductors with a degenerate valence band: Dark and bright exciton states. Phys. Rev. B.

[76] Brovelli S., Schaller R.D., Crooker S.A., García-Santamaría F., Chen Y., Viswanatha R., Hollingsworth J.A., Htoon H., Klimov V.I. (2011). Nano-engineered electron–hole exchange interaction controls exciton dynamics in core–shell semiconductor nanocrystals. Nat. Commun..

[77] Kigel A., Brumer M., Maikov G.I., Sashchiuk A., Lifshitz E. (2009). Thermally activated photoluminescence in lead selenide colloidal quantum dots. Small.

[78] Shabaev A., Hellberg C.S., Efros A.L. (2013). Efficiency of multiexciton generation in colloidal nanostructures. Acc. Chem. Res..

[79] Vaxenburg R., Lifshitz E., Efros A.L. (2013). Suppression of auger-stimulated efficiency droop in nitride-based light emitting diodes. Appl. Phys. Lett..

[80] Delerue C., Allan G., Pijpers J.J.H., Bonn M. (2010). Carrier multiplication in bulk and nanocrystalline semiconductors: Mechanism, efficiency, and interest for solar cells. Phys. Rev. B.

[81] Padilha L.A., Stewart J.T., Sandberg R.L., Bae W.K., Koh W.-K., Pietryga J.M., Klimov V.I. (2013). Aspect ratio dependence of auger recombination and carrier multiplication in PbSe nanorods. Nano Lett..

[82] Trinh M.T., Limpens R., de Boer W.D.A.M., Schins J.M., Siebbeles L.D.A., Gregorkiewicz T. (2012). Direct generation of multiple excitons in adjacent silicon nanocrystals revealed by induced absorption. Nat. Photonics.

[83] An J.M., Franceschetti A., Dudiy S.V., Zunger A. (2006). The peculiar electronic structure of PbSe quantum dots. Nano Lett..

[84] An J.M., Franceschetti A., Zunger A. (2007). The excitonic exchange splitting and radiative lifetime in PbSe quantum dots. Nano Lett..

[85] Liu H., Guyot-Sionnest P. (2010). Photoluminescence lifetime of lead selenide colloidal quantum dots. J. Phys. Chem. C.

[86] Ouyang J., Vincent M., Kingston D., Descours P., Boivineau T., Zaman M.B., Wu X., Yu K. (2009). Noninjection, one-pot synthesis of photoluminescent colloidal homogeneously alloyed CdSes quantum dots. J. Phys. Chem. C.

[87] Zhong X., Han M., Dong Z., White T.J., Knoll W. (2003). Composition-tunable Zn*_x_*Cd_1–*x*_Se nanocrystals with high luminescence and stability. J. Am. Chem. Soc..

[88] Bailey R.E., Nie S. (2003). Alloyed semiconductor quantum dots: Tuning the optical properties without changing the particle size. J. Am. Chem. Soc..

[89] Smith D.K., Luther J.M., Semonin O.E., Nozik A.J., Beard M.C. (2010). Tuning the synthesis of ternary lead chalcogenide quantum dots by balancing precursor reactivity. ACS Nano.

[90] Maikov G.I., Vaxenburg R., Sashchiuk A., Lifshitz E. (2010). Composition-tunable optical properties of colloidal IV–VI quantum dots, composed of core/shell heterostructures with alloy components. ACS Nano.

[91] Wang X., Ren X., Kahen K., Hahn M.A., Rajeswaran M., Maccagnano-Zacher S., Silcox J., Cragg G.E., Efros A.L., Krauss T.D. (2009). Non-blinking semiconductor nanocrystals. Nature.

[92] Li L., Daou T.J., Texier I., Kim Chi T.T., Liem N.Q., Reiss P. (2009). Highly luminescent CuInS_2_/ZnS core/shell nanocrystals: Cadmium-free quantum dots for* in vivo* imaging. Chem. Mater..

[93] Osovsky R., Cheskis D., Kloper V., Sashchiuk A., Kroner M., Lifshitz E. (2009). Continuous-wave pumping of multiexciton bands in the photoluminescence spectrum of a single CdTe-CdSe core-shell colloidal quantum dot. Phys. Rev. Lett..

[94] Oron D., Kazes M., Banin U. (2007). Multiexcitons in type-II colloidal semiconductor quantum dots. Phys. Rev. B.

[95] Vaxenburg R., Lifshitz E. (2012). Alloy and heterostructure architectures as promising tools for controlling electronic properties of semiconductor quantum dots. Phys. Rev. B.

[96] Abel K.A., Qiao H., Young J.F., van Veggel F.C.J.M. (2010). Four-fold enhancement of the activation energy for nonradiative decay of excitons in PbSe/CdSe core/shell* versus* PbSe colloidal quantum dots. J. Phys. Chem. Lett..

[97] Lambert K., Geyter B.D., Moreels I., Hens Z. (2009). PbTe|CdTe core|shell particles by cation exchange, a HR-TEM study. Chem. Mater..

[98] Lee D.C., Robel I., Pietryga J.M., Klimov V.I. (2010). Infrared-active heterostructured nanocrystals with ultralong carrier lifetimes. J. Am. Chem. Soc..

[99] Bartnik A.C., Efros A.L., Koh W.K., Murray C.B., Wise F.W. (2010). Electronic states and optical properties of PbSe nanorods and nanowires. Phys. Rev. B.

[100] De Geyter B., Justo Y., Moreels I., Lambert K., Smet P.F., Van Thourhout D., Houtepen A.J., Grodzinska D., de Mello Donega C., Meijerink A. (2010). The different nature of band edge absorption and emission in colloidal PbSe/CdSe core/shell quantum dots. ACS Nano.

[101] Allan G., Delerue C. (2004). Confinement effects in PbSe quantum wells and nanocrystals. Phys. Rev. B.

[102] Zhou S., Dong L., Popov S., Friberg A.T. (2013). Radiative properties of carriers in CdSe-CdS core-shell heterostructured nanocrystals of various geometries. J. Eur. Opt. Soc. Rap. Publ..

[103] Cragg G.E., Efros A.L. (2009). Suppression of auger processes in confined structures. Nano Lett..

[104] Wei S.-H., Zunger A. (1997). Electronic and structural anomalies in lead chalcogenides. Phys. Rev. B.

[105] Knapp R.A. (1963). Photoelectric properties of lead sulfide in the near and vacuum ultraviolet. Phys. Rev..

[106] Preier H. (1979). Recent advances in lead-chalcogenide diode lasers. Appl. Phys. A.

[107] Zhang Y., Dai Q., Li X., Liang J., Colvin V.L., Wang Y., Yu W.W. (2011). PbSe/CdSe and PbSe/CdSe/ZnSe hierarchical nanocrystals and their photoluminescence. Langmuir.

[108] Yanover D., Čapek R.K., Rubin-Brusilovski A., Vaxenburg R., Grumbach N., Maikov G.I., Solomeshch O., Sashchiuk A., Lifshitz E. (2012). Small-sized PbSe/PbS core/shell colloidal quantum dots. Chem. Mater..

[109] Dai Q., Wang Y., Li X., Zhang Y., Pellegrino D.J., Zhao M., Zou B., Seo J., Wang Y., Yu W. (2009). Size-dependent composition and molar extinction coefficient of PbSe semiconductor nanocrystals. ACS Nano.

[110] Moreels I., Lambert K., de Muynck D., Vanhaecke F., Poelman D., Martins J., Allan G., Hens Z. (2007). Composition and size-dependent extinction coefficient of colloidal PbSe quantum dots. Chem. Mater..

[111] Murray C.B., Shouheng S., Gaschler W., Doyle H., Betley T.A., Kagan C.R. (2001). Colloidal synthesis of nanocrystals and nanocrystal superlattices. IBM J. Res. Dev..

[112] Houtepen A.J., Koole R., Vanmaekelbergh D., Meeldijk J., Hickey S.G. (2006). The hidden role of acetate in the PbSe nanocrystal synthesis. J. Am. Chem. Soc..

[113] Jasieniak J., Califano M., Watkins S.E. (2011). Size-dependent valence and conduction band-edge energies of semiconductor nanocrystals. ACS Nano.

[114] Grinbom G.A., Saraf M., Saguy C., Bartnik A.C., Wise F., Lifshitz E. (2010). Density of states in a single PbSe/PbS core-shell quantum dot measured by scanning tunneling spectroscopy. Phys. Rev. B.

[115] Garrett M.D., Dukes A.D., McBride J.R., Smith N.J., Pennycook S.J., Rosenthal S.J. (2008). Band edge recombination in CdSe, CdS and CdS_x_Se_1−x_ alloy nanocrystals observed by ultrafast fluorescence upconversion: The effect of surface trap states. J. Phys. Chem. C.

[116] Huang Y.H., Cheng C.L., Chen T.T., Chen Y.F., Tsen K.T. (2007). Studies of stokes shift in In_x_Ga_1–X_N alloys. J. Appl. Phys..

[117] Swafford L.A., Weigand L.A., Bowers M.J., McBride J.R., Rapaport J.L., Watt T.L., Dixit S.K., Feldman L.C., Rosenthal S.J. (2006). Homogeneously alloyed CdS_x_Se_1–x_ nanocrystals: Synthesis, characterization, and composition/size-dependent band gap. J. Am. Chem. Soc..

[118] Hyun B.-R., Zhong Y.-W., Bartnik A.C., Sun L., Abruña H.D., Wise F.W., Goodreau J.D., Matthews J.R., Leslie T.M., Borrelli N.F. (2008). Electron injection from colloidal PbS quantum dots into titanium dioxide nanoparticles. ACS Nano.

[119] Tang J., Brzozowski L., Barkhouse D.A.R., Wang X., Debnath R., Wolowiec R., Palmiano E., Levina L., Pattantyus-Abraham A.G., Jamakosmanovic D. (2010). Quantum dot photovoltaics in the extreme quantum confinement regime: The surface-chemical origins of exceptional air- and light-stability. ACS Nano.

[120] Krauss T.D., Wise F.W. (1997). Raman-scattering study of exciton-phonon coupling in PbS nanocrystals. Phys. Rev. B.

[121] Grodzińska D., Evers W.H., Dorland R., van Rijssel J., van Huis M.A., Meijerink A., de Mello Donegá C., Vanmaekelbergh D. (2011). Two-fold emission from the s-shell of PbSe/CdSe core/shell quantum dots. Small.

[122] Harbold J.M., Wise F.W. (2007). Photoluminescence spectroscopy of PbSe nanocrystals. Phys. Rev. B.

[123] Lifshitz E., Brumer M., Kigel A., Sashchiuk A., Bashouti M., Sirota M., Galun E., Burshtein Z., Le Quang A.Q., Ledoux-Rak I. (2006). Air-stable PbSe/PbS and PbSe/PbSe_x_S_1–x_ core/shell nanocrystal quantum dots and their applications. J. Phys. Chem. B.

[124] An J.M., Califano M., Franceschetti A., Zunger A. (2008). Excited-state relaxation in PbSe quantum dots. J. Chem. Phys..

[125] Lifshitz E., Vaxenburg R., Maikov G.I., Rubin-Brusilovski A., Yanover D., Tilchin J., Sashchiuk A. (2012). The influence of alloy composition on the electronic properties of IV–VI core/shell colloidal heterostructures. Isr. J. Chem..

[126] Moreels I., Lambert K., Smeets D., de Muynck D., Nollet T., Martins J.C., Vanhaecke F., Vantomme A., Delerue C., Allan G. (2009). Size-dependent optical properties of colloidal PbS quantum dots. ACS Nano.

[127] Oron D., Aharoni A., de Mello Donega C., van Rijssel J., Meijerink A., Banin U. (2009). Universal role of discrete acoustic phonons in the low-temperature optical emission of colloidal quantum dots. Phys. Rev. Lett..

[128] Gurioli M., Vinattieri A., Colocci M., Deparis C., Massies J., Neu G., Bosacchi A., Franchi S. (1991). Temperature dependence of the radiative and nonradiative recombination time in GaAs/Al_x_Ga_1–x_ as quantum-well structures. Phys. Rev. B.

[129] Chun Hsiung W., Tzung T.C., Yang F.C., Mei L.H., Chih W.L., Pi T.C. (2008). Recombination dynamics in CdTe/CdSe type-II quantum dots. Nanotechnology.

[130] Pandey A., Guyot-Sionnest P. (2008). Slow electron cooling in colloidal quantum dots. Science.

[131] Cirloganu C.M., Padilha L.A., Lin Q., Makarov N.S., Velizhanin K.A., Luo H., Robel I., Pietryga J.M., Klimov V.I. (2014). Enhanced carrier multiplication in engineered quasi-type-II quantum dots. Nat. Commun..

[132] Neo D.C.J., Cheng C., Stranks S.D., Fairclough S.M., Kim J.S., Kirkland A.I., Smith J.M., Snaith H.J., Assender H.E., Watt A.A.R. (2014). Influence of shell thickness and surface passivation on PbS/CdS core/shell colloidal quantum dot solar cells. Chem. Mater..

[133] Grumbach N., Rubin-Brusilovski A., Maikov G.I., Tilchin E., Lifshitz E. (2013). Manipulation of carrier–Mn^2+^ exchange interaction in CdTe/CdSe colloidal quantum dots by controlled positioning of Mn^2+^ impurities. J. Phys. Chem. C.

[134] Chikan V. (2011). Challenges and prospects of electronic doping of colloidal quantum dots: Case study of CdSe. J. Phys. Chem. Lett..

[135] Ma G. (2013). Background-free* in vivo* time domain optical molecular imaging using colloidal quantum dots. ACS Appl. Mater. Interfaces.

[136] Pichaandi J., van Veggel F.C.J.M. (2014). Near-infrared emitting quantum dots: Recent progress on their synthesis and characterization. Coord. Chem. Rev..

